# Low carbohydrate ketogenic diets reduce cardiovascular risk factor levels in obese or overweight patients with T2DM: A meta-analysis of randomized controlled trials

**DOI:** 10.3389/fnut.2022.1092031

**Published:** 2022-12-13

**Authors:** Wei Luo, Jin Zhang, Dan Xu, Yao Zhou, Zhen Qu, Qin Yang, Qiujv Lv

**Affiliations:** Department of Endocrinology, People's Hospital of Leshan, Leshan, Sichuan, China

**Keywords:** low-carbohydrate ketogenic diet, overweight, obesity, T2DM, cardiovascular risk

## Abstract

**Background:**

The purpose of this meta-analysis was to explore the effects of low-carbohydrate ketogenic diets on cardiovascular risk factors in overweight or obese patients. However, there are limited literature data about effects of low-carbohydrate ketogenic diets on cardiovascular risk factors in obese or overweight patients.

**Methods:**

We systematically searched PubMed, EMBASE, Web of Science, OVID, and Cochrane Library databases (last updated in September 2022) for randomized controlled trials (RCTs) which recruited overweight or obesity patients on ketogenic diets in order to control cardiovascular risk factors (blood glucose, weight, and lipids). The overall effect size for continuous variables was expressed as a weighted standardized mean difference (SMD) with a confidence interval of 95%. Considering type 2 diabetes mellitus (T2DM) status at baseline, subgroup analyses were performed when appropriate, based on T2DM comorbidity among patients. The effect model was selected according to heterogeneity.

**Results:**

We finally selected 21 studies. Low carbohydrate ketogenic diets exerted a greater impact on cardiovascular risk factors in obese/ overweight patients with T2DM when compared with those on non-ketogenic diets, with lower fasting plasma glucose (FPG) (SMD, −0.75; *P* < 0.001) and hemoglobin A1c (HbA1c) (SMD, −0.53; *P* < 0.001) levels identified. Low-carbohydrate ketogenic diets significantly reduced body mass index (BMI) (SMD, −2.27; *P* = 0.032), weight (SMD, −6.72; *P* < 0.001), and waist circumference (SMD, −4.45; *P* = 0.003) in obese/ overweight patients with T2DM. Also, ketogenic diets improved lipid profiles in these patients; triglyceride (TG) (SMD, −0.32; *P* = 0.013) levels were lowered and high density lipoprotein (HDL) showed an upward trend with the *P*-value close to statistically significant level (SMD, −0.32; *P* = 0.052). In general, irrespective of diabetic status at baseline, ketogenic diets were more effective in reducing TG (SMD, −0.2; *P* = 0.02) and increasing HDL (SMD, 0.11; *P* = 0.03) levels when compared with non-ketogenic diets.

**Conclusions:**

Low-carbohydrate ketogenic diets effectively improved cardiovascular risk factors (blood glucose, weight, and lipids) in obese/ overweight patients, especially those with T2DM when compared with non-ketogenic diets.

## 1. Introduction

In both developing and developed countries, obesity levels continue to grow with ~38 and 20% of the world's adult population predicted to be overweight and obese by 2030 (1), respectively. On a global level, obesity is one of the most serious health issues (2, 3). Moreover, a recent study by Pi-Sunyer et al. (3) suggested that obesity was a major potential risk factor for chronic diseases, such as cardiovascular disease, diabetes mellitus (DM), cerebrovascular disease, metabolic syndrome, and cancer, thereby increasing social, medical, and economic burdens. Importantly, studies have also shown that normal weight reduces metabolic syndrome prevalence and all-cause and cardiovascular mortality when compared with obese or overweight individuals (4). The benefits of weight loss are not only related to blood sugar control, but also to several cardiovascular risk factors, such as blood pressure, high-density lipoprotein (HDL), total cholesterol (TC), triglyceride (TG), low-density lipoprotein (LDL), and uric acid (UA) levels (5). Dietary therapy for weight loss is commonly recommended in clinical practice (6).

Previous studies have proposed many different dietary approaches, including low-carbohydrate ketogenic diets which includes ~60% fat intake, 25% protein intake, and 15% carbohydrate intake, but without limiting caloric intake (7, 8). Ketogenic diets with low carbohydrate content may mimic starvation in the body and generate ketosis. Thus, the glucose-based energy model is replaced by the ketone body-based energy model, which requires fat to promote catabolism and reduce fat synthesis, while gluconeogenesis increases energy expenditure (9). To this end, insoluble TGs are converted to water-soluble ketone bodies. Therefore, ketone bodies can be eliminated by the body *via* urine excretion, taking away energy (10). In addition, increased ketone also bodies suppress appetite (11). Such diets are widely reported as being effective for weight loss and glycemic control (12). A recent review showed that low-carbohydrate diets reduced postprandial glucose levels and exerted cardioprotective effects (13). A meta-analysis of 14 studies showed that a ketogenic diet improved the metabolic parameters in overweight or obese patients when compared with a low-fat diet, it was particularly effective for metabolic parameters related to blood glucose, weight, and lipid control in patients with previous diabetes (14); however, Leow et al. (15) showed that low-carbohydrate diets potentially exacerbated lipid profiles in obese patients. The evidence supporting ketogenic diets is currently limited and the diet's potential risks are real (12). Therefore, appropriateness and potential risks of low-carbohydrate ketogenic diets in obese patients remain controversial. To address this issue, we investigated the clinical effects of ketogenic diets in overweight or obese patients when compared with non-ketogenic diets by assessing changes in cardiovascular risk factors related to glycemic, weight, UA, blood pressure, and lipid levels.

## 2. Methods

### 2.1. Data sources and search strategy

The following databases were systematically searched: Pubmed, EMBASE, OVID, Web of Science, and Cochrane Library (last updated September 2022). A combined Medical Subject Heading and free word search strategy was adopted. Key words included: ketogenic diet, obesity, overweight, abdominal obesity, type 2 diabetes, blood sugar, insulin resistance, hyperlipidemia, lipid metabolism, hyperuricemia, metabolic syndrome, cardiovascular risk factors, randomized controlled trials (RCTs), and clinical trials. Additionally, to avoid missing publications, bibliographies from previous reviews were also screened.

### 2.2. Study selection and selection criteria

Inclusion criteria: (1) randomized clinical trials (RCTs) published up to September 2022 which investigated the effects of ketogenic diets on metabolic parameters in overweight or obese patients; (2) adequate original data which could be obtained from the original research, and (3) full original research texts.

Exclusion criteria: case-controls studies, cohort studies, cross-sectional studies, animal experiments, reviews, commentaries, editorials, and case reports. Study titles and summaries were independently assessed by two reviewers using the inclusion criteria. For potentially eligible studies, two reviewers assessed full texts. In case of disagreement, matters were settled through consultation and a consensus reached by the corresponding authors (XD and JZ).

### 2.3. Data extraction and quality assessment

Two reviewers independently extracted data from eligible articles into tables. Extracted data included: author's name, publication year, country, study population, mean age, gender, sample size, BMI, follow-up duration, diet type, and metabolic parameters of ketogenic and non-ketogenic diet groups (Table 1 and Supplementary Table S1).

**Table 1:** Participant characteristics in selected studies.

			**Experience/Control**					
**Author**	**Country**	**Gender**	**Diet**	**Sample**	**Age**	**BMI (kg/m2)**	**Follow-up (weeks)**	**Population**
Vazquez and Kazi (16)	USA	Both	LCKD/LCD	8/8	44 ± 3/44 ± 5	41 ± 5/37 ± 6	4	Obesity/Overweight
Yancy et al. (17)	UAS	Both	LCKD/LFD	59/60	45.3 ± 9.5/44.1 ± 8.7	34.56 ± 5.2/33.9 ± 5.3	24	Overweight
Westman et al. (18)	USA	Both	LCKD/LFD	59/60	44.2 ± 10.1/45.6 ± 9.0	34.6 ± 4.9/34.1 ± 5.1	24	Overweight
Yancy et al. (19)	USA	Both	LCKD/LFD	27/12	43.8 ± 10.2/42.8 ± 7.3	36.0 ± 4.9/36.0 ± 5.7	24	Obesity
Westman et al. (20)	USA	Both	LCKD/LCD	21/29	51.2 ± 6.1/50.0 ± 8.4	37.8 ± 6.7/37.9 ± 6.0	24	Obesity with T2DM
Yancy et al. (21)	USA	Both	LCKD/LFD	57/65	54.5 ± 9.7/53.2 ± 8.9	39.5 ± 6.9/39.3 ± 7.2	48	Obesity/Overweight
Jabekk et al. (22)	Norway	Female	LCKD/UD	8/8	/	32.9 ± 4.5 /31.7 ± 4.2	10	Obesity/Overweight
Partsalaki et al. (23)	Greece	Both	LCKD/LCD	21/17	13.6 ± 2.5/12.3 ± 2.7	30.8 ± 8.1/28.0 ± 4.2	24	Obesity/Overweight
Saslow et al. (24)	USA	Both	VLCKD/MCCR	15/18	64.8 ± 7.7/55.1 ± 13.5	/	12	Obesity/Overweight with T2DM
Goday et al. (25)	Spain	Both	VLCKD/LCD	45/44	54.89 ± 8.81/54.17 ± 7.97	33.25 ± 1.52/32.88 ± 1.6	16	Obesity with T2DM
Colica et al. (26)	Italy	Both	VLCKD/Placebo	20/20	45.40 ± 14.20	30.45 ± 2.64	3	Obesity/Overweight
Myette-Côté et al. (27)	Columbia	Both	LCKD/LCD	16/16	64 ± 8	34 ± 8	0.57	Obesity/Overweight with T2DM
Sun et al. (28)	China	Female	LCKD/UD	15/15	21.6 ± 3.9/20.9 ± 3.7	24.8 ± 3.2/25 ± 2.9	4	Obesity/Overweight
Perissiou et al. (29)	UK	Both	LCKD/UD	31/33	35 ± 6/34 ± 8	31.2 ± 3/30.8 ± 4	8	Obesity
Lodi et al. (30)	Italy	Female	LCKD/MD	15/15	/	28.4 ± 2.4/27 ± 1.9	1.4	Overweight
Cunha et al. (31)	USA	Both	VLCKD/LCD	20/19	40.3 ± 11.3	37.1 ± 4.28 /34.84 ± 4.33	8	Obesity/Overweight
Hall et al. (32)	USA	Both	LCKD/LFD	19/20	29.9 ± 6.4	27.8 ± 5.9	2	Overweight
Vidic et al. (33)	Serbia	Male	LCKD/NKD	9/9	42.7 ± 1.5	26.75 ± 2.7	8	Obesity/Overweight
Paoli et al. (34)	Italy	Male	LCKD/WD	9/10	26.22 ± 5.09/31.67 ± 10.39	26.97 ± 1.86/26.66 ± 2.04	8	Obesity/Overweight
Li (9)	China	Both	LCKD/DDCD	24/29	36.50 ± 13.67/37.10 ± 14.02	29.04 ± 5.81/29.75 ± 6.07	12	Obesity/Overweight with T2DM
Wu et al. (35)	China	Both	LCKD/HBD	19/50	31 ± 9/34 ± 9	32.2 ± 4.2/31.4 ± 3.5	12	Obesity

RCT, Randomized controlled trial; LCKD, Low carbohydrate ketogenic diet; VLCKD, Very low carbohydrate ketogenic diet; LFD, Low fat diet; NKD, Non ketogenic diet; MCCR, Medium carbohydrate, calorie restriction; UD, Regular diet; WD, Western-style food; BMI, body mass index; LCD, Low calorie diet; HD, High carbohydrate diet; MD, Mediterranean diet; CRD, Energy restricted balanced diet; DDCD, diabetes diet control diet; HBD, Hypocaloric balanced diet.

Using the Cochrane Collaboration Risk of Bias Tool, RCT methodological quality was assessed by two reviewers (Supplementary Figure S2); areas were assessed with a low, medium, or high risk of deviation rather than as a range of scores. Differences were resolved by consultation and a consensus reached by corresponding authors (XD and ZJ).

### 2.4. Statistical analysis

The overall effect size for continuous variables was expressed as a weighted standardized mean difference (SMD) with a confidence interval (CI) of 95%. Post-treatment metabolic parameters were compared between patients on ketogenic and non-ketogenic diets. Considering T2DM status at baseline and to minimize potential heterogeneity sources, subgroup analyses were performed when appropriate, based on T2DM comorbidity among patients. Heterogeneity was assessed using *I*^2^ and Cochran's Q tests, with *I*^2^ > 50% and *p* < 0.01 indicating significant heterogeneity. If significant, the random-effect model was adopted; otherwise, the fixed-effect model was applied. Two-tailed *P* < 0.05 values were considered statistically significant. All analyses were performed using STATA version 14.0. Sensitivity analyses were performed by omitting one study at a time. Egger's linear regression was used to test publication bias, which was evaluated by the trim-and-fill method.

## 3. Results

### 3.1. Study selection, characteristics, and quality assessment

This meta-analysis included 21 RCTs (9, 16–35) involving 1,074 subjects. The process and literature screening results are shown (Supplementary Figure S1). The basic characteristics and results from selected studies are shown (Table 1). Raw extracted data are shown (Supplementary Table S1).

### 3.2. The effects of low-carbohydrate ketogenic diets on glucose metabolism

**Figure 1:**
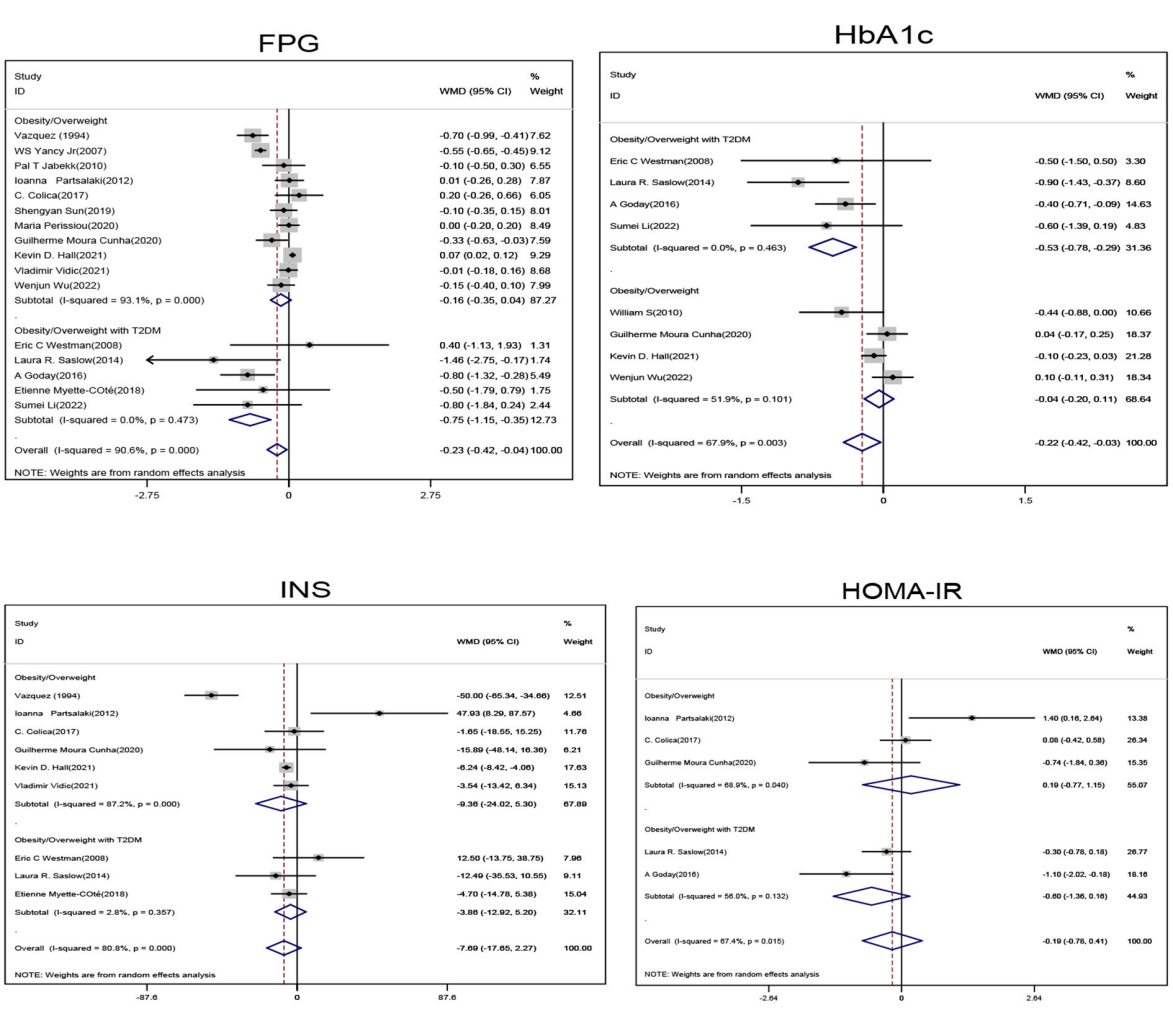
Forest plot showing weighted mean difference and 95% confidence intervals (CIs) for low carbohydrate ketogenic diet effects on glucose metabolism. FPG, Fasting blood glucose; HbA1c, Glycosylated hemoglobin; INS, Insulin; HOMA-IR, Homeostatic model assessment.

Subgroup analysis of obese/overweight patients with T2DM showed that when compared with the non-ketogenic diet group, FPG (SMD, −0.75; 95% CI, −1.15 to −0.35; *I*^2^ = 0.00%, Z = 3.7; *P* < 0.001) and HbA1c levels (SMD, −0.53; 95% CI, −0.78 to −0.29; *I*^2^ = 0.00%, Z = 4.24; *P* < 0.001) were significantly decreased in the ketogenic diet group. Subgroup analysis of non-diabetic patients showed that the glycemic index was not significantly decreased. In general, ketogenic diets were more effective in reducing FPG (SMD, −0.23; 95% CI, −0.42 to −0.04, *I*^2^ = 90.6%, Z = 2.38, *P* = 0.017) and HbA1c (SMD, −0.22; 95% CI, −0.42 to −0.03, *I*^2^= 67.9%, Z = 2.24, *P* = 0.025) levels when compared with non-ketogenic diets (Figure 1).

### 3.3. The effects of low-carbohydrate ketogenic diets on lipid metabolism

**Figure 2:**
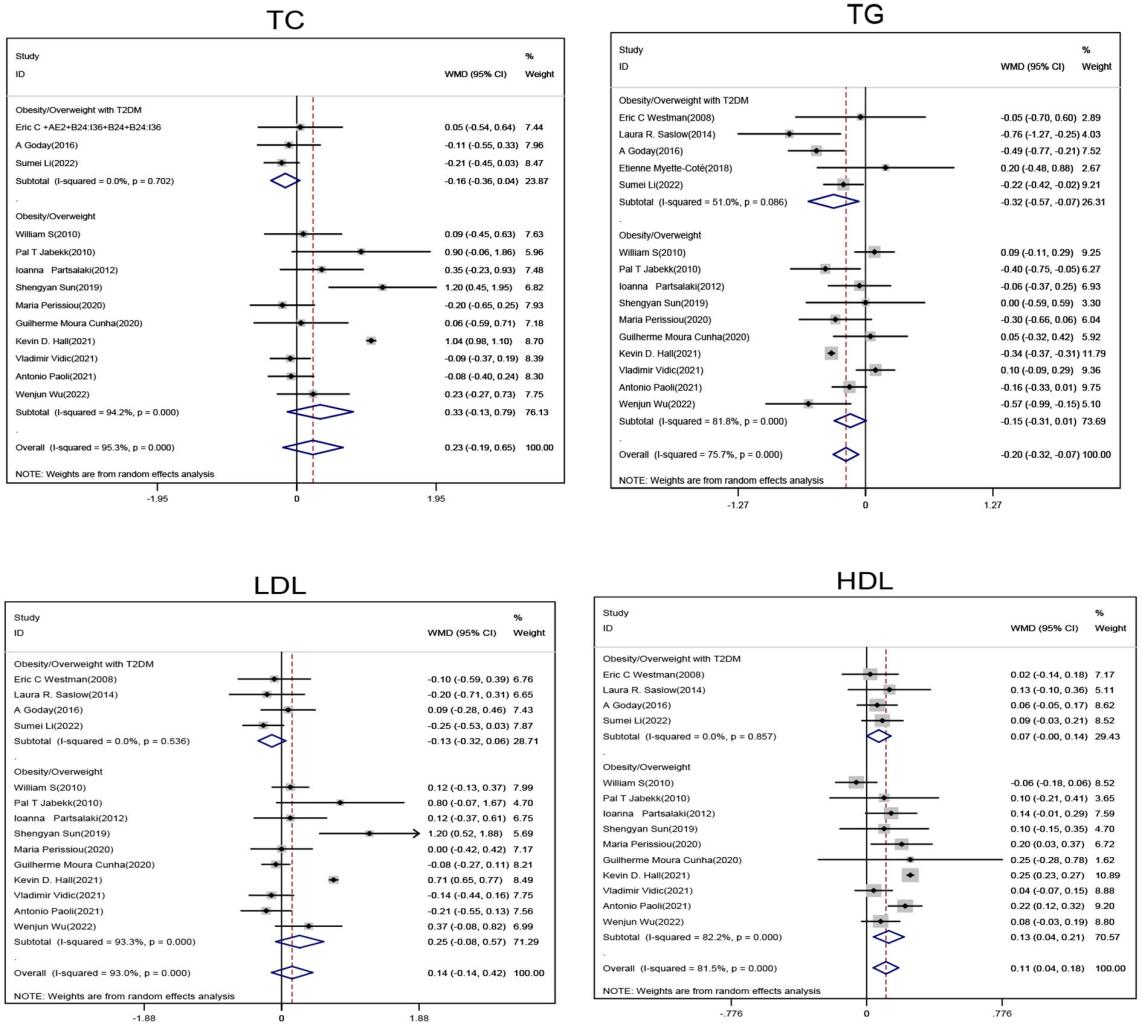
Forest plot showing weighted mean difference and 95% confidence intervals (CIs) for low carbohydrate ketogenic diet effects on lipid metabolism. TC, Total cholesterol; TG, Triglyceride; LDL, Low density lipoprotein; HDL, High density lipoprotein.

Subgroup analysis of obese/overweight patients with T2DM showed that when compared with the non-ketogenic diet group, TG levels (SMD, −0.32; 95% CI,− 0.57 to−0.07; I^2^ = 51%, Z = 2.48; *P* = 0.013) were significantly decreased in the ketogenic diet group, and HDL levels showed an upward trend and a *P*-value close to statistically significant level (SMD, 0.07; 95% CI, −0.00 to 0.14; *I*^2^ = 0.00%, Z = 1.95; *P* = 0.052). Subgroup analyses of obese/overweight patients without T2DM showed that when compared with the non-ketogenic diet, HDL levels were significantly higher in the ketogenic diet group (SMD, 0.13,95% CI, 0.04 to 0.21, *I*^2^ = 82.2%, Z = 2.85, *P* = 0.004), and TG levels showed a downward trend and a *P*-value close to statistically significant level (SMD, −0.15; 95% CI, −0.31 to 0.01; *I*^2^ = 81.1%, Z = 1.88; *P* = 0.06). In general, ketogenic diets were more effective in reducing TG (SMD, −0.2; 95% CI, −0.32 to −0.07, *I*^2^ = 75.7%, Z = 3.03, *P* = 0.02) and increasing HDL (SMD, 0.11; 95% CI, 0.04 to 0.18, *I*^2^ = 81.5%, Z = 2.98, *P* = 0.03) levels when compared with non-ketogenic diets. However, ketogenic diets had no significant effect on changes on TC and LDL levels (Figure 2).

### 3.4 The effects of low-carbohydrate ketogenic diets on weight control

**Figure 3:**
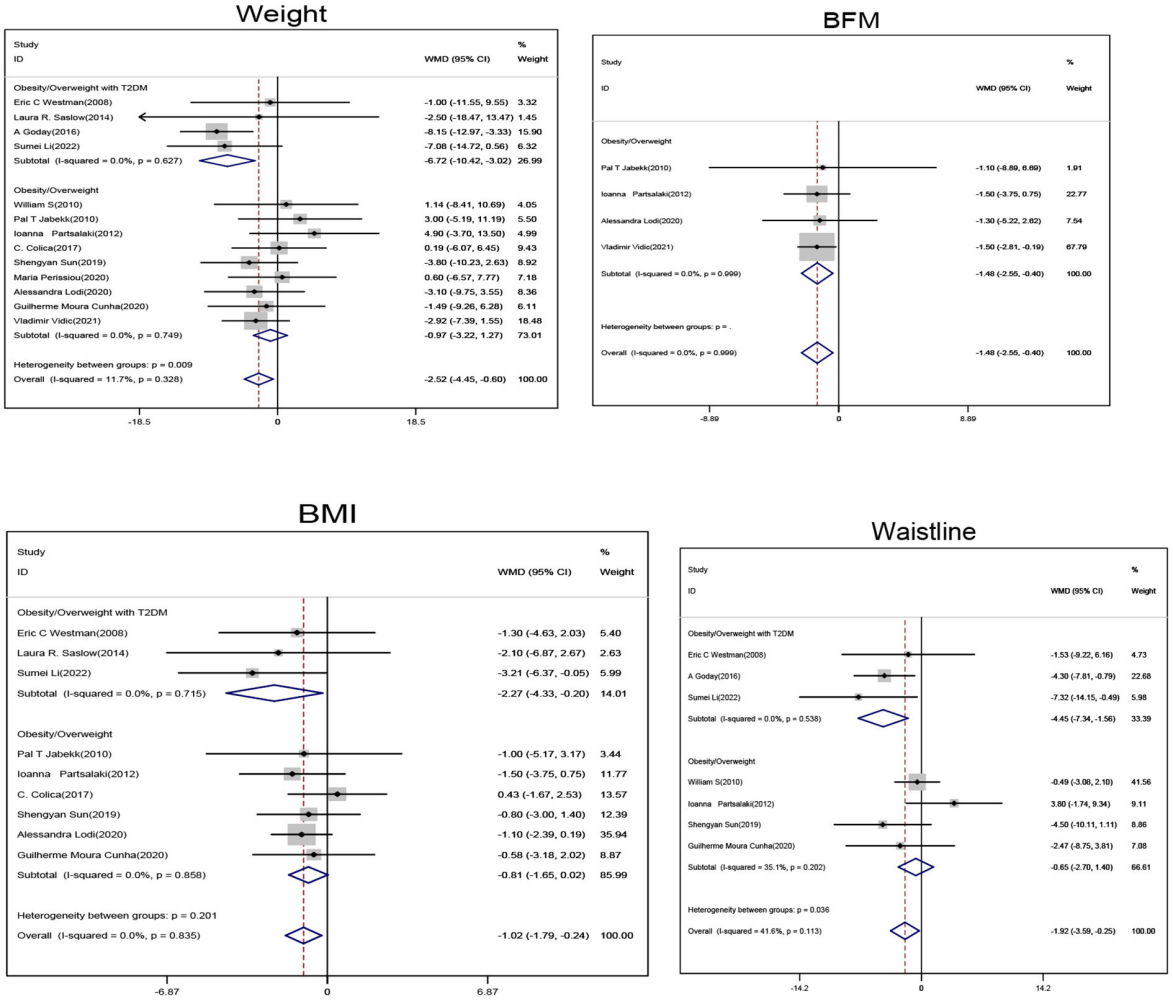
Forest plot showing weighted mean difference and 95% confidence intervals (CIs) for low carbohydrate ketogenic diet effects on weight control. BFM, Body fat volume; BMI, Body mass index.

Subgroup analysis of obese/overweight patients with T2DM showed that when compared with the non-ketogenic diet group, subjects in the ketogenic diet group showed significant reductions in weight (SMD, −6.72; 95% CI, −10.42 to −3.02; *I*^2^ = 0.00%, Z = 3.56; P < 0.001), BMI (SMD, −2.27; 95% CI, −4.33 to −0.2; *I*^2^ = 0.00%, Z = 2.15; P = 0.032) and waistline circumference (SMD, −4.45; 95% CI, −7.34 to −1.56; *I*^2^ = 0.00%, Z = 3.01; *P* = 0.003). Subgroup analysis of obese/overweight patients without T2DM showed that when compared with non-ketogenic diet, subject's body fat mass (BFM) (SMD, −1.48; 95% CI, −2.55 to −0.4; *I*^2^ = 0.00%, Z = 2.69; *P* = 0.007) was significantly decreased in the ketogenic diet group. In general, weight (SMD, −2.52; 95% CI, −4.45 to −0.6; *I*^2^ = 11.7%, Z = 2.58; *P* = 0.01), BMI (SMD, −1.02; 95% CI, −1.79 to −0.24; *I*^2^ = 0.00%, Z = 2.57; *P* = 0.01), and waistline circumference (SMD, −1.92; 95% CI, −3.59 to −0.25; *I*^2^ = 41.6%, Z = 2.25; *P* = 0.025) were significantly decreased in the ketogenic diet group (Figure 3).

### 3.5. The effects of low-carbohydrate ketogenic diets on renal related metabolic indices and blood pressure

**Figure 4:**
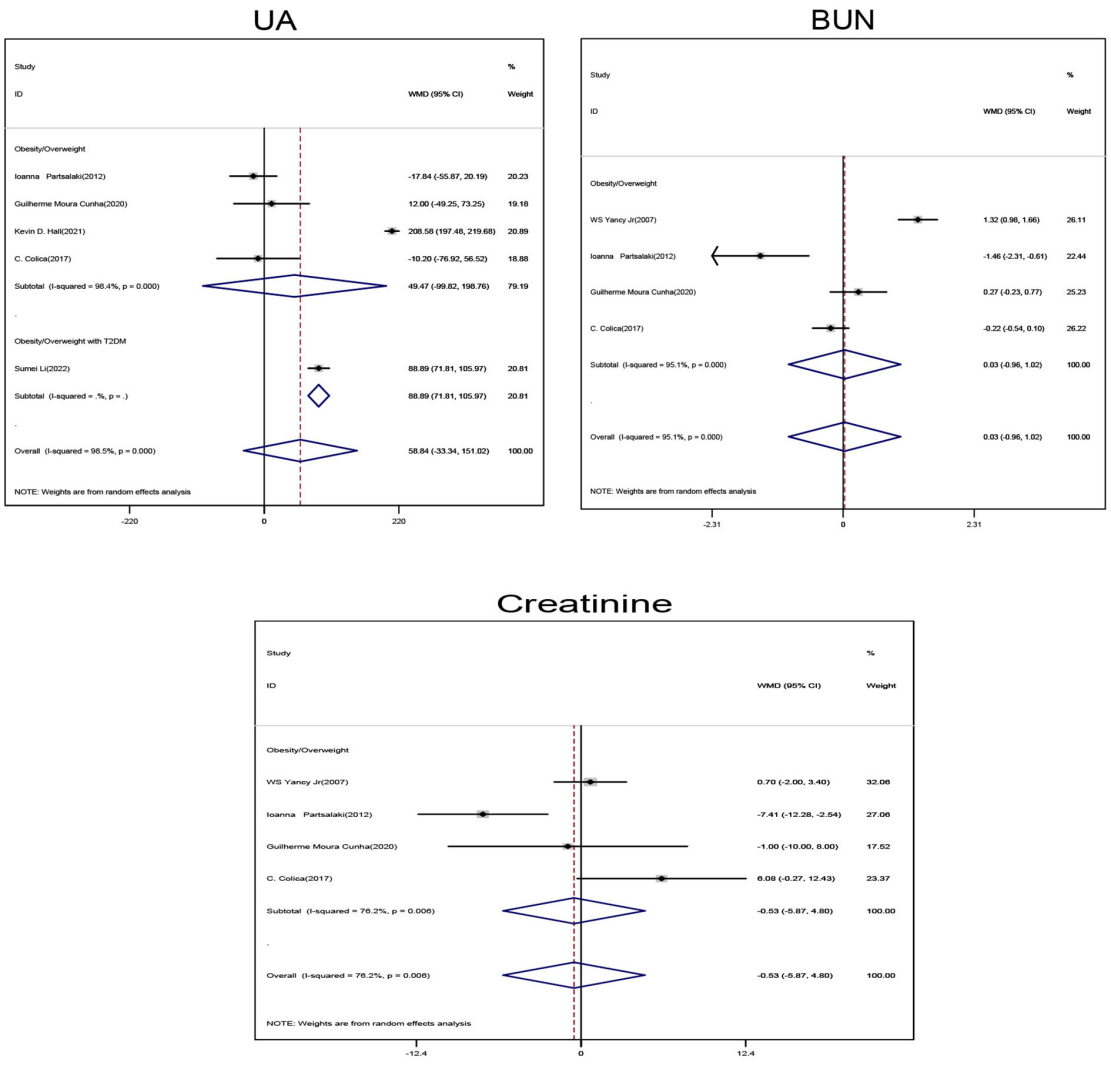
Forest plot showing weighted mean difference and 95% confidence intervals (CIs) for low carbohydrate ketogenic diet effects on kidney related functions. UA, Uric acid; BUN, Urea nitrogen.

Obese/overweight patients on ketogenic diet showed no statistically significant changes in Uric acid (UA), blood urea nitrogen (BUN), and creatinine levels when compared with those on non-ketogenic diet (Figure 4).

**Figure 5:**
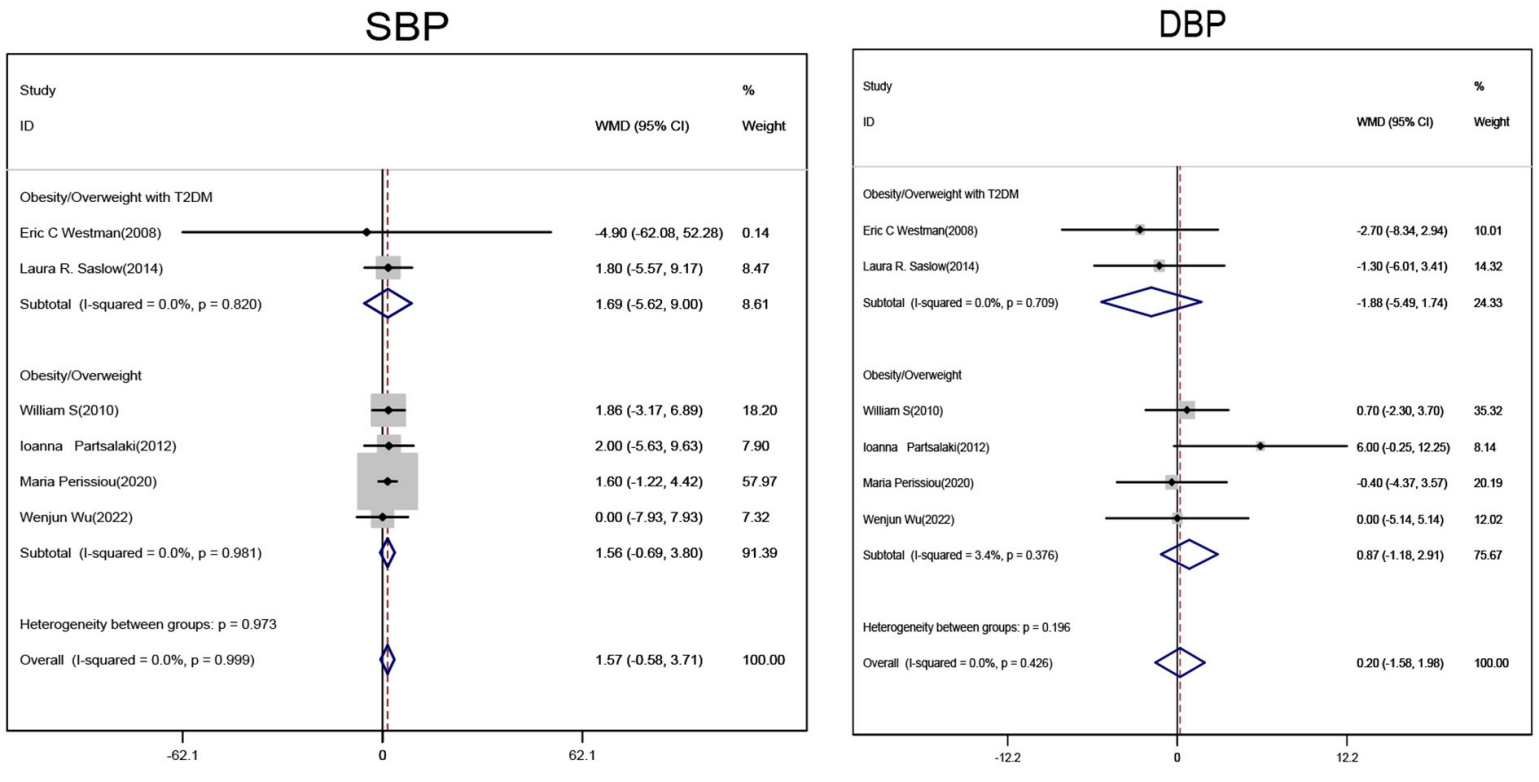
Forest plot showing weighted mean difference and 95% confidence intervals (CIs) for low carbohydrate ketogenic diet effects on blood pressure. SBP, Systolic pressure; DBP, Diastolic pressure.

Subgroup analyses of obese/overweight patients with or without T2DM on ketogenic diet showed no statistically significant changes in systolic and diastolic blood pressure levels when compared with non-ketogenic diet (Figure 5).

### 3.6. Sensitivity analyses, publication bias, and trim-and-fill evaluations

In sensitivity analyses, suspected heterogeneity sources were identified and removed when data were pooled (Supplementary Figures S3–S9).

Egger linear regression analyses were used to test for publication bias. Ketogenic diets showed significant publication bias toward TC, LDL, HDL, and UA (Egger Test's *P* = 0.004, 0.006, 0.002, and 0.048, respectively) (Supplementary Tables S3–S6).

These results showed that publication bias had no effects on our observations, because the *P*-values of combined effect values before and after trim-and-fill analyses were not reversed. We observed no significant differences in heterogeneity and merging results before and after trim and fill analyses, suggesting our stable results (Supplementary Table S7).

## 4. Discussion

In this meta-analysis of 21 RCTs which included 1,074 participants, we investigated the efficacy of ketogenic diets when compared with non-ketogenic diets toward cardiovascular risk factor control in overweight or obese patients. Our results showed: (1) In obese/overweight patients with T2DM, low-carbohydrate ketogenic diets significantly improved FPG and HbA1c levels and showed diets were more effective in controlling blood glucose. (2) In obese/overweight patients with T2DM and overall patients, low-carbohydrate ketogenic diets significantly decreased TG levels and increased HDL levels. (3) In obese/overweight patients with T2DM, after ketogenic diet intervention, weight, BMI, and waist circumference indices were significantly decreased, while BFM was also significantly decreased in non-diabetic patients. Our findings suggested that low-carbohydrate ketogenic diets were more beneficial dietary options for obese/overweight T2DM patients and improved cardiovascular risk factors related to glycemic, weight, and lipid control.

Concomitant with increased global obesity levels, cardiovascular disease levels have similarly increased (36). Obesity is also a major risk factor for insulin resistance and T2DM (37). An insulin resistance status is often associated with ectopic lipid accumulation, especially in the liver and skeletal muscle (38). Obesity and diabetes often co-exist in the same patient (37). Clear associations have been identified between obesity and cardiovascular disease incidence, including hypertension, coronary artery disease, heart failure, and sudden death. Many individuals with insulin resistance, the leading cause of T2DM, tend to have higher TG and lower HDL levels (39–41). Obesity, diabetes and dyslipidemia are all risk factors for cardiovascular disease (39–41).

The importance of a proper balanced diet can-not be underestimated when managing and preventing chronic diseases. The American Diabetes Association recommends a combination of physical activity, diet management, and medication intake to control blood glucose, weight, and other abnormal metabolic factors. Ketogenic diets have many health benefits for obese/overweight patients with T2DM (11, 42). These diets provide energy *via* fat oxidation, and when the body experiences extreme hunger or limited carbohydrate levels, ketone bodies are produced and released into the circulation *via* fatty acid conversion in the liver (43). Nutritional ketosis is distinct to severe pathological diabetic ketosis; blood ketone bodies are maintained in the 0.5–3.0 mmol/l range, blood glucose is decreased, and no nutritional ketosis symptoms are present (44).

Our meta-analysis confirmed that ketogenic diets significantly reduced FPG and HbA1c levels in obese/overweight patients with T2DM. A possible mechanism for this health benefit could be extreme dietary sugar restriction which reduces intestinal monosaccharide absorption, leading to lower blood glucose levels and reduced glucose fluctuations. Considerable evidence now suggests the effectiveness of ketogenic diets in regulating glucose metabolism (14, 45). HbA1c effectively reflects blood glucose control in the previous 2–3 months in patients with diabetes. When HbA1c levels drop, the risks of myocardial infarction and microvascular complications are reportedly decreased (21, 46).

Ketogenic diets not only improve glucose metabolism, but also improve lipid metabolism (in particular for obese/overweight patients with T2DM), and decrease TG and increase HDL levels so as, to improve abnormal blood lipid levels. Dyslipidemia is lipotoxic to cells and causes and/or exacerbates insulin resistance. The condition is characterized by elevated TG and free fatty acids (FFA) levels. Increased FFA is an independent risk factor for insulin resistance and may increase cardiovascular disease risks (47, 48). Also, in considering that low HDL and high TG levels are independent risk factors for insulin resistance and cardiovascular disease (49), our analyses suggest a cardioprotective effect for ketogenic diets in patients with diabetes; ketogenic diets appear to significantly affect TG and HDL levels in these individuals. Therefore, improved dyslipidemia is not only beneficial for regulating insulin sensitivity, but also protects against cardiovascular disease (49, 50).

In obese/overweight individuals with T2DM, ketogenic diets significantly reduced weight, BMI, and waist circumference. Hall et al. suggested that diets high in carbohydrates were prone to generating obesity as they tended to increase insulin production (51). Insulin-guided energy distribution is stored in adipose tissue in the form of fat, rather than being oxidized by metabolically active tissue, and is believed to result in a perceived state of hunger within cells (51). In response, hunger and appetite increase and metabolism is suppressed, thereby promoting a positive energy balance associated with obesity development (51). Ketogenic diets trigger gluconeogenesis and ketogenesis in the liver, producing ketone bodies from glucose-substituting fatty acids. Ketone body production usually occurs when endogenous glucose production is depleted and lowers insulin levels in the blood, which further limits fat and glucose storage in the body (50, 52). A clear association exists between obesity and cardiovascular disease incidence; however, with improved weight control, cardiovascular disease incidence and mortality is also decreased (53).

Our RCT meta-analysis had the following advantages: firstly, as an RCT meta-analysis, we investigated the effects of ketogenic diets on cardiovascular risk factors (glycemic control, weight loss, and lipid control) in various non-ketogenic diets. Accordingly, obese/overweight patients with T2DM were more likely to benefit from ketogenic diets in terms of weight loss, improved blood glucose levels, and lipid control. Secondly, the meta-analysis included a large number of participants (*n* = 1,074). Thirdly, comprehensive sensitivity analyses, publication bias, and trim and fill evaluations indicated the reliability of our results. Fourthly, all selected studies were RCTs, which further increased the reliability of our conclusions.

However, our meta-analysis had the following limitations: firstly, we combined RCT ketogenic diets with different control diets. Secondly, cardiovascular risk factors were used to explore the metabolic effects of ketogenic diets, including blood glucose levels, weight, and lipid composition, rather than to assess cardiovascular disease incidence and mortality in patients. However, the effects of ketogenic diets on cardiovascular risk factors could help validate the effects of low-carbohydrate ketogenic diets on clinical cardiovascular event endpoints. Finally, heterogeneity was evident in our data and was possibly due to differences in control diets and follow-up durations across the 21 studies. However, our comprehensive sensitivity analysis, publication bias, and trim and fill evaluation indicated the reliability of our observations.

In conclusion, this is a large, comprehensive and RCTs meta-analysis designed to explore the effect of low-carbohydrate ketogenic diets on cardiovascular risk factors in obese/overweight patients. Our results showed that low-carbohydrate ketogenic diets were more effective than control diets in improving cardiovascular risk factors (body weight, blood glucose, and lipid levels) in obese/overweight patients especially those with T2DM. But there remains a requirement for further prospective studies, to determine the long-term effects of low-carbohydrate ketogenic diets on cardiovascular risk markers in obese/overweight populations, and to determine their impact on cardiovascular event endpoints in these populations.

## Data availability statement

The original contributions presented in the study are included in the article/Supplementary material, further inquiries can be directed to the corresponding authors.

## Author contributions

WL and JZ had full access to all of the data in the study and takes responsibility for the integrity of the data and the accuracy of the data analysis. Study concept and design: WL, DX, and JZ. Acquisition of data: YZ and QY. Drafting of the manuscript: WL. Statistical analysis: WL, ZQ, and JZ. Study supervision: DX and JZ. Analysis and interpretation of data and critical revision of the manuscript for important intellectual content: All authors.
