# Supplementary material for: Low carbohydrate ketogenic diets reduce cardiovascular risk factor levels in obese or overweight patients with T2DM: A meta-analysis of randomized controlled trials
Source: Front Nutr. 2022 Dec 13;9:1092031. doi: 10.3389/fnut.2022.1092031 (PMC9792675; doi:10.3389/fnut.2022.1092031)
Supplement: Supplementary file 1 [file Data_Sheet_1.PDF]

Glycometabolism	FPG(mmol/l)		LCKD	NKD	N	
		Vazquez(1994)	3.8± 0.3	4.5±0.3	8/8	Obesity/Overweight
		WS Yancy Jr(2007)	4.67±0.1	5.22±0.16	27/12	Obesity
		Eric C Westman(2008)	8.78±2.77	8.38±2.65	21/29	Obesity with T2DM
		Pal T Jabekk(2010)	5.0±0.3	5.1±0.5	8/8	Obesity/Overweight
		Ioanna Partsalaki(2012)	4.51±0.29	4.5±0.5	21/17	Obesity/Overweight
		Laura R. Saslow(2014)	6.29±0.85	7.75±2.63	15/18	Obesity/Overweight with T2DM
		A Goday(2016)	6.05±1.13	6.85±1.35	45/44	Obesity with T2DM
		C. Colica(2017)	4.40±0.54	4.20±0.89	20/20	Obesity/Overweight
		Étienne Myette-Côté(2018)	7.6±1.7	8.1±2.0	16/16	Obesity/Overweight with T2DM
		Shengyan Sun(2019)	4.6±0.4	4.7±0.3	15/15	Overweight
		Maria Perissiou(2020)	5.4±0.4	5.4±0.4	31/33	Obesity
		Guilherme Moura Cunha(2020)	4.76±0.43	5.09±0.51	20/19	Obesity
		Kevin D. Hall(2021)	4.74±0.08	4.67±0.08	19/20	Obesity
		Vladimir Vidic(2021)	5.02±0.19	5.03±0.18	9/9	Obesity/Overweight
		Sumei Li(2022)	7.62±1.69	8.42±2.17	24/29	Obesity/Overweight with T2DM
		Wenjun Wu(2022)	4.86±0.47	5.01±0.51	19/50	Obesity

HbA1c(%)		LCKD	NKD	N	
	Eric C Westman(2008)	7.3±1.5	7.8±2.1	21/29	Obesity with T2DM
	William S(2010)	5.9±1.15	6.34±1.34	57/65	Obesity/Overweight
	Laura R. Saslow(2014)	6.0±0.3	6.9±1.1	15/18	Obesity/Overweight with T2DM
	A Goday(2016)	6.0±0.7	6.4±0.8	45/44	Obesity with T2DM
	刘秀(2020)	6.52±1.76	8.28±1.91	49/49	Overweight with T2DM
	Guilherme Moura Cunha(2020)	5.41±0.36	5.37±0.31	20/19	Obesity/Overweight
	Kevin D. Hall(2021)	5.0±0.2	5.1±0.2	19/20	Overweight
	Sumei Li(2022)	7.82±1.43	8.42±1.51	24/29	Obesity/Overweight with T2DM
	Wenjun Wu(2022)	5.2±0.4	5.1±0.4	19/50	Obesity

INS(pmol/l)		LCKD	NKD	N	
	Vazquez(1994)	43±7	93±21	8/8	Obesity/Overweight
	Eric C Westman(2008)	99.94±47.89	87.44±45.11	21/29	Obesity with T2DM
	Ioanna Partsalaki(2012)	125.01±63.89	77.08±60.42	21/17	Obesity/Overweight
	Laura R. Saslow(2014)	64.54±33.31	77.03±34	15/18	Obesity/Overweight with T2DM
	C. Colica(2017)	49.89±23.05	51.54±30.92	20/20	Obesity/Overweight
	Étienne Myette-Côté(2018)	26.0± 12.8	30.7± 16.1)	16/16	Obesity/Overweight with T2DM
	Guilherme Moura Cunha(2020)	88.14±51.73	104.03±51.01	20/19	Obesity
	Kevin D. Hall(2021)	51.36± 3.47	57.60±3.47	19/20	Overweight
	Vladimir Vidic(2021)	40.67±9.79	44.21±11.53	9/9	Overweight
	Sumei Li(2022)	40.38±9.54	84.21±10.79	24/29	Obesity/Overweight with T2DM

HOMA-IR		LCKD	NKD	N	
	Ioanna Partsalaki(2012)	3.6±2.0	2.2±1.9	21/17	Obesity/Overweight
	Laura R. Saslow(2014)	1.3 ±0.6	1.6 ±0.8	15/18	Obesity/Overweight with T2DM
	A Goday(2016)	3.5 ±1.9	4.6 ±2.5	45/44	Obesity with T2DM
	C. Colica(2017)	1.44 ± 0.75	1.36 ± 0.86	20/20	Obesity/Overweight
	Guilherme Moura Cunha(2020)	2.70 ±1.58	3.44 ±1.90	20/19	Obesity/Overweight

Lipid metabolism	TC(mmol/l)		LCKD	NKD	N	
		Eric C Westman(2008)	4.83±0.93	4.78±1.18	21/29	Obesity with T2DM
		William S(2010)	4.62±1.96	4.53±0.68	57/65	Obesity/Overweight
		Pal T Jabekk(2010)	5.7±1.2	4.8±0.7	8/8	Obesity/Overweight
		Ioanna Partsalaki(2012)	4.4±0.85	4.05±0.94	21/17	Obesity/Overweight
		A Goday(2016)	4.84±1.2	4.95±0.88	45/44	Obesity with T2DM
		Shengyan Sun(2019)	6.1±1.3	4.9±0.7	15/15	Obesity/Overweight
		Maria Perissiou(2020)	4.6±1.0	4.8±0.8	31/33	Obesity
		Guilherme Moura Cunha(2020)	4.48±1.34	4.42±0.64	20/19	Obesity
		Kevin D. Hall(2021)	4.16±0.1	3.12±0.1	19/20	Obesity/Overweight
		Vladimir Vidic(2021)	4.43±0.30	4.52±0.30	9/9	Obesity/Overweight
		Antonio Paoli(2021)	4.85±0.26	4.93±0.43	9/10	Obesity/Overweight
		Sumei Li(2022)	4.02±0.43	4.23±0.47	24/29	Obesity/Overweight with T2DM
		Wenjun Wu(2022)	5.12±0.96	4.89±0.90	19/50	Obesity

TG(mmol/l)		LCKD	NKD	N	
	Eric C Westman(2008)	1.62±0.87	1.67±1.45	21/29	Obesity with T2DM
	William S(2010)	1.39±0.51	1.3±0.59	57/65	Obesity/Overweight
	Pal T Jabekk(2010)	0.9±0.3	1.3±0.4	8/8	Obesity/Overweight
	Ioanna Partsalaki(2012)	0.83±0.35	0.89±0.57	21/17	Obesity/Overweight
	Laura R. Saslow(2014)	1.14±0.45	1.9±0.99	15/18	Obesity/Overweight with T2DM
	A Goday(2016)	1.29±0.65	1.78±0.69	45/44	Obesity with T2DM
	Étienne Myette-Côté(2018)	2.1±1.2	1.9±0.7	16/16	Obesity/Overweight with T2DM
	Shengyan Sun(2019)	1.1±0.6	1.1±1.0	15/15	Obesity with T2DM
	Maria Perissiou(2020)	1±0.9	1.3±0.5	31/33	Obesity
	Guilherme Moura Cunha(2020)	1.29±0.62	1.24±0.55	20/19	Obesity
	Kevin D. Hall(2021)	0.71±0.05	1.05±0.05	19/20	Obesity/Overweight
	Vladimir Vidic(2021)	0.95±0.26	0.85±0.13	9/9	Obesity/Overweight
	Antonio Paoli(2021)	1.11±0.22	1.27±0.15	9/10	Obesity/Overweight
	Sumei Li(2022)	1.44±0.26	1.66±0.46	24/29	Obesity/Overweight with T2DM
	Wenjun Wu(2022)	1.29±0.65	1.86±1.10	19/50	Obesity

LDL(mmol/l)		LCKD	NKD	N	
	Eric C Westman(2008)	2.77±0.68	2.87±1.09	21/29	Obesity with T2DM
	William S(2010)	2.94±0.76	2.82±0.62	57/65	Obesity/Overweight
	Pal T Jabekk(2010)	3.5±1.1	2.7±0.6	8/8	Obesity/Overweight
	Ioanna Partsalaki(2012)	2.72±0.69	2.6±0.83	21/17	Obesity/Overweight
	Laura R. Saslow(2014)	2.25±0.87	2.45±0.56	15/18	Obesity/Overweight with T2DM
	A Goday(2016)	2.86±0.99	2.77±0.77	45/44	Obesity with T2DM
	Shengyan Sun(2019)	4.2±1.2	3.0±0.6	15/15	Obesity/Overweight
	Maria Perissiou(2020)	2.9±0.9	2.9±0.8	31/33	Obesity
	Guilherme Moura Cunha(2020)	1.23±0.18	1.31±0.38	20/19	Obesity
	Kevin D. Hall(2021)	2.38±0.09	1.67±0.1	19/20	Obesity/Overweight
	Vladimir Vidic(2021)	2.45±0.25	2.59±0.39	9/9	Obesity/Overweight
	Antonio Paoli(2021)	2.79±0.26	3±0.48	9/10	Obesity/Overweight
	Sumei Li(2022)	2.34±0.45	2.59±0.58	24/29	Obesity/Overweight with T2DM
	Wenjun Wu(2022)	3.27±0.89	2.90±0.75	19/50	Obesity

HDL((mmol/l))		LCKD	NKD	N	
	Eric C Westman(2008)	1.28±0.3	1.26±0.26	21/29	Obesity with T2DM
	William S(2010)	1.05±0.24	1.11±0.41	57/65	Obesity/Overweight
	Pal T Jabekk(2010)	1.5±0.4	1.4±0.2	8/8	Obesity/Overweight
	Ioanna Partsalaki(2012)	1.27±0.26	1.13±0.20	21/17	Obesity/Overweight
	Laura R. Saslow(2014)	1.32±0.38	1.19±0.29	15/18	Obesity/Overweight with T2DM
	A Goday(2016)	1.41±0.29	1.35±0.26	45/44	Obesity with T2DM
	Shengyan Sun(2019)	1.7±0.3	1.6±0.4	15/15	Obesity/Overweight
	Maria Perissiou(2020)	1.4±0.4	1.2±0.3	31/33	Obesity
	Guilherme Moura Cunha(2020)	2.81±1.06	2.56±0.55	20/19	Obesity
	Kevin D. Hall(2021)	1.21±0.03	0.96±0.03	19/20	Obesity/Overweight
	Vladimir Vidic(2021)	1.40±0.11	1.36±0.12	9/9	Obesity/Overweight
	Antonio Paoli(2021)	1.55±0.08	1.33±0.13	9/10	Obesity/Overweight
	Sumei Li(2022)	1.21±0.23	1.12±0.20	24/29	Obesity/Overweight with T2DM
	Wenjun Wu(2022)	1.13±0.20	1.05±0.22	19/50	Obesity

Weight control	Weight（kg)		LCKD	NKD	N	
		Eric C Westman(2008)	97.3±17.6	98.3±20.3	21/29	Obesity with T2DM
		William S(2010)	112.63±17.81	111.49±34.37	57/65	Obesity/Overweight
		Pal T Jabekk(2010)	90.0±8.8	87.0±7.9	8/8	Obesity/Overweight
		Ioanna Partsalaki(2012)	65.7±14.2	60.8±12.8	21/17	Obesity/Overweight
		Laura R. Saslow(2014)	94.6 ±23.3	97.1±23.3	15/18	Obesity/Overweight with T2DM
		A Goday(2016)	76.8±9.1	84.95±13.6	45/44	Obesity with T2DM
		C. Colica(2017)	77.62± 12.37	77.43± 7.12	20/20	Obesity/Overweight
		Shengyan Sun(2019)	62.3 ±6.7	66.1±10.8	15/15	Obesity/Overweight
		Maria Perissiou(2020)	83.4±16	82.8±13	31/33	Obesity
		Alessandra Lodi(2020)	71.9±12.06	75±5.2	15/15	Overweight
		Guilherme Moura Cunha(2020)	89.85±10.94	91.34±13.61	20/19	Obesity/Overweight
		Vladimir Vidic(2021)	81.17±4.35	84.09±5.38	9/9	Obesity/Overweight
		Sumei Li(2022)	70.26±14.79	77.34±13.28	24/29	Obesity/Overweight with T2DM

Body fat volume (kg)		LCKD	NKD	N	
	Pal T Jabekk(2010)	38.4±9.1	39.5±6.6	8/8	Obesity/Overweight
	Ioanna Partsalaki(2012)	26.0±8.1	21.8±8.2	21/17	Overweight
	Alessandra Lodi(2020)	27±6.3	28.3±4.5	15/15	Overweight
	Vladimir Vidic(2021)	11.2±1.2	12.7±1.6	9/9	Overweight

BMI(kg/m2)		LCKD	NKD	N	
	Eric C Westman(2008)	33.9±5.8	35.2±6.1	21/29	Obesity with T2DM
	Pal T Jabekk(2010)	31.0±4.5	32.0±4.0	8/8	Obesity/Overweight
	Ioanna Partsalaki(2012)	24.8±3	26.3±3.9	21/17	Obesity/Overweight
	Laura R. Saslow(2014)	34.3±7.4	36.4±6.4	15/18	Obesity/Overweight with T2DM
	A Goday(2016)	27.9 ±1.8	31.0 ±2.2	45/44	Obesity with T2DM
	C. Colica(2017)	26.54±4.14	26.11± 2.42	20/20	Obesity/Overweight
	Shengyan Sun(2019)	24.0±2.7	24.8 ±3.4	15/15	Obesity/Overweight
	Alessandra Lodi(2020)	25.9±1.9	27±1.7	15/15	Overweight
	Guilherme Moura Cunha(2020)	33.55±3.73	34.13±4.49	20/19	Obesity/Overweight
	Sumei Li(2022)	26.21±5.74	29.42±5.97	24/29	Obesity/Overweight with T2DM

Waist(cm)		LCKD	NKD	N	
	Eric C Westman(2008)	106.17±13.46	107.7±14	21/29	Obesity with T2DM
	William S(2010)	116.53±6.13	117.02±8.42	57/65	Obesity/Overweight
	Ioanna Partsalaki(2012)	93.0±7.8	89.2±9.3	21/17	Obesity/Overweight
	A Goday(2016)	96.1±7.6	100.4 ±9.2	45/44	Obesity with T2DM
	Shengyan Sun(2019)	74.0±6.2	78.5±9.2	15/15	Obesity/Overweight
	Guilherme Moura Cunha(2020)	103.00±9.68	105.47±10.31	20/19	Obesity/Overweight
	Sumei Li(2022)	99.24±14.58	106.56±9.78	24/29	Obesity/Overweight with T2DM

Kidney function	Uric acid(umol/l)		LCKD	NKD	N	
		Ioanna Partsalaki(2012)	291.45±59.48	309.29±59.48	21/17	Obesity/Overweight
		Guilherme Moura Cunha(2020)	510 ±104	498± 91	20/19	Obesity
		Kevin D. Hall(2021)	632.48±17.68	424.3±17.68	19/20	Obesity/Overweight
		C. Colica(2017)	307.8± 114.6	318 ± 100.2	20/20	Obesity/Overweight
		Sumei Li(2022)	467.43±35.67	378.54±25.79	24/29	Obesity/Overweight with T2DM

Urea nitrogen(mmol/l)		LCKD	NKD	N	
	WS Yancy Jr(2007)	6.21±0.33	4.89±0.56	27/12	Obesity
	Ioanna Partsalaki(2012)	8.64±1.43	10.1±1.25	21/17	Obesity/Overweight
	Guilherme Moura Cunha(2020)	2.82±0.89	2.55±0.68	20/19	Obesity
	C. Colica(2017)	1.59±0.34	1.81±0.64	20/20	Obesity/Overweight

Creatinine(umol/l)		LCKD	NKD	N	
	WS Yancy Jr(2007)	61.0±2.8	60.3±4.4	27/12	Obesity
	Ioanna Partsalaki(2012)	45.97±7.62	53.38±7.62	21/17	Obesity/Overweight
	Guilherme Moura Cunha(2020)	75±9	76±18	20/19	Obesity
	C. Colica(2017)	63.64±11.49	57.56±8.84	20/20	Obesity/Overweight

Blood pressure	SBP(mmHg)		LCKD	NKD	N	
		Eric C Westman(2008)	142.9±76.9	147.8± 128.5	21/29	Obesity with T2DM
		William S(2010)	129.16±10.46	127.3±17.4	57/65	Obesity/Overweight
		Ioanna Partsalaki(2012)	108±13	106±11	21/17	Obesity/Overweight
		Laura R. Saslow(2014)	136.0±9.8	134.2± 11.8	15/18	Obesity/Overweight with T2DM
		Maria Perissiou(2020)	119.3±7	117.7±4	31/33	Obesity
		Wenjun Wu(2022)	125±16	125±12	19/50	Obesity

DBP(mmHg)		LCKD	NKD	N	
	Eric C Westman(2008)	75.8±10.9	78.5±8.7	21/29	Obesity with T2DM
	William S(2010)	84.87±4.77	84.17±11.24	57/65	Obesity/Overweight
	Ioanna Partsalaki(2012)	68±8	62±11	21/17	Obesity/Overweight
	Laura R. Saslow(2014)	78.9±6.5	80.2±7.3	15/18	Obesity/Overweight with T2DM
	Maria Perissiou(2020)	78.6±9	79±7	31/33	Obesity
	Wenjun Wu(2022)	72±10	72±9	19/50	Obesity

Table S1 Outcomes and covariates of included studies.

Egger's test

Std_Eff	Coef.	Std. Err.	t	P>|t|	[95% Conf.	Interval]
slope	.0074105	.0866225	0.09	0.933	-.1783764	.1931973
bias	-1.544686	1.078484	-1.43	0.174	-3.857806	.7684326

A FPG

Egger's test

Std_Eff	Coef.	Std. Err.	t	P>|t|	[95% Conf.	Interval]
slope	.1025801	.1183978	0.87	0.420	-.1871288	.3922891
bias	-1.993843	.9350071	-2.13	0.077	-4.281723	.294037

B HbA1c

Egger's test

Std_Eff	Coef.	Std. Err.	t	P>|t|	[95% Conf.	Interval]
slope	-6.376936	3.217255	-1.98	0.088	-13.98454	1.230664
bias	-.113072	1.037028	-0.11	0.916	-2.565255	2.33911

C INS

Egger's test

Std_Eff	Coef.	Std. Err.	t	P>|t|	[95% Conf.	Interval]
slope	-.213738	.8340309	-0.26	0.814	-2.867997	2.440521
bias	.104503	2.426121	0.04	0.968	-7.616497	7.825502

D HOAM-IR

Table S2 Egger's test for the effect of ketogenic diet on glucose metabolism.

Egger's test

Std_Eff	Coef.	Std. Err.	t	P>|t|	[95% Conf.	Interval]
slope	1.104016	.1228926	8.98	0.000	.8335311	1.374501
bias	-4.317416	1.199882	-3.60	0.004	-6.958339	-1.676494

A TC（**There is publication bias. The stability of the results will be proved by trim and fill analysis in the future.**）

Egger's test

Std_Eff	Coef.	Std. Err.	t	P>|t|	[95% Conf.	Interval]
slope	-.346212	.0338603	-10.22	0.000	-.4193627	-.2730613
bias	1.107827	.5916175	1.87	0.084	-.170285	2.385939

B TG

Egger's test

Std_Eff	Coef.	Std. Err.	t	P>|t|	[95% Conf.	Interval]
slope	.7458418	.1020838	7.31	0.000	.5234203	.9682632
bias	-3.545565	1.058068	-3.35	0.006	-5.850898	-1.240232

C LDL (**There is publication bias. The stability of the results will be proved by trim and fill analysis in the future.**)

Egger's test

Std_Eff	Coef.	Std. Err.	t	P>|t|	[95% Conf.	Interval]
slope	.2639886	.017882	14.76	0.000	.225027	.3029502
bias	-2.176099	.5532213	-3.93	0.002	-3.381464	-.970733

D HDL (**There is publication bias. The stability of the results will be proved by trim and fill analysis in the future.**)

Table S3 Egger's test for the effect of ketogenic diet on glucose metabolism.

Egger's test

Std_Eff	Coef.	Std. Err.	t	P>|t|	[95% Conf.	Interval]
slope	-8.220918	3.314179	-2.48	0.031	-15.51538	-.9264592
bias	1.683683	.9377442	1.80	0.100	-.3802778	3.747644

A Weight

Egger's test

Std_Eff	Coef.	Std. Err.	t	P>|t|	[95% Conf.	Interval]
slope	-1.591903	.0383864	-41.47	0.001	-1.757066	-1.426739
bias	.1218843	.0349678	3.49	0.073	-.0285699	.2723386

B BFM

Egger's test

Std_Eff	Coef.	Std. Err.	t	P>|t|	[95% Conf.	Interval]
slope	-2.094438	.8631575	-2.43	0.041	-4.084882	-.1039929
bias	5.018613	2.692332	1.86	0.099	-1.189916	11.22714

C BMI

Egger's test

Std_Eff	Coef.	Std. Err.	t	P>|t|	[95% Conf.	Interval]
slope	-.3478873	3.137351	-0.11	0.916	-8.412704	7.716929
bias	-.7512664	1.390428	-0.54	0.612	-4.325475	2.822942

.

D Waistline

Table S4 Egger's test for the effect of ketogenic diet on weight control.

Egger's test

Std_Eff	Coef.	Std. Err.	t	P>|t|	[95% Conf.	Interval]
slope	-3.590509	1.37727	-2.61	0.080	-7.973598	.7925801
bias	11.97554	3.685616	3.25	0.048	.2462635	23.70481

A UA (**There is publication bias. The stability of the results will be proved by trim and fill analysis in the future.**)

Egger's test

Std_Eff	Coef.	Std. Err.	t	P>|t|	[95% Conf.	Interval]
slope	-6.116186	3.058616	-2.00	0.184	-19.27635	7.043977
bias	17.61629	8.566366	2.06	0.176	-19.2418	54.47439

B BUN

Egger's test

Std_Eff	Coef.	Std. Err.	t	P>|t|	[95% Conf.	Interval]
slope	.252553	6.542279	0.04	0.973	-27.8966	28.40171
bias	-.3082219	2.979505	-0.10	0.927	-13.128	12.51155

C Creatinine

Table S5 Egger's test for the effect of ketogenic diet on kidney related functions.

Egger's test

Std_Eff	Coef.	Std. Err.	t	P>|t|	[95% Conf.	Interval]
slope	1.968428	.4277984	4.60	0.010	.7806695	3.156187
bias	-.1762533	.1596073	-1.10	0.331	-.6193941	.2668876

A SBP

Egger's test

Std_Eff	Coef.	Std. Err.	t	P>|t|	[95% Conf.	Interval]
slope	-.6201728	3.979732	-0.16	0.884	-11.66968	10.42934
bias	.3796471	1.786138	0.21	0.842	-4.579466	5.338761

B DBP

Table S6 Egger's test for the effect of ketogenic diet on blood pressure

.

```
Meta-analysis

       |  Pooled      95% CI         Asymptotic      No. of
Method |     Est   Lower   Upper  z_value  p_value   studies
-------+----------------------------------------------------
Fixed  |   0.813   0.758   0.869   28.630    0.000      13
Random |   0.228  -0.190   0.646    1.071    0.284

Test for heterogeneity: Q= 254.427 on 12 degrees of freedom (p= 0.000)
Moment-based estimate of between studies variance =  0.521

Trimming estimator: Linear
Meta-analysis type: Random-effects model

iteration |  estimate    Tn    # to trim     diff
----------+--------------------------------------
    1     |    0.228     39         0          91
    2     |    0.228     39         0           0

Note: no trimming performed; data unchanged

Filled
Meta-analysis

       |  Pooled      95% CI         Asymptotic      No. of
Method |     Est   Lower   Upper  z_value  p_value   studies
-------+----------------------------------------------------
Fixed  |   0.813   0.758   0.869   28.630    0.000      13
Random |   0.228  -0.190   0.646    1.071    0.284

Test for heterogeneity: Q= 254.427 on 12 degrees of freedom (p= 0.000)
Moment-based estimate of between studies variance =  0.521
```

A TC

```
Meta-analysis

       |  Pooled      95% CI         Asymptotic      No. of
Method |     Est   Lower   Upper  z_value  p_value   studies
-------+----------------------------------------------------
Fixed  |   0.506   0.455   0.557   19.624    0.000      14
Random |   0.143  -0.138   0.424    1.000    0.317

Test for heterogeneity: Q= 185.128 on 13 degrees of freedom (p= 0.000)
Moment-based estimate of between studies variance =  0.241

Trimming estimator: Linear
Meta-analysis type: Random-effects model

iteration |  estimate    Tn    # to trim     diff
----------+--------------------------------------
    1     |    0.143     45         0         105
    2     |    0.143     45         0           0

Note: no trimming performed; data unchanged

Filled
Meta-analysis

       |  Pooled      95% CI         Asymptotic      No. of
Method |     Est   Lower   Upper  z_value  p_value   studies
-------+----------------------------------------------------
Fixed  |   0.506   0.455   0.557   19.624    0.000      14
Random |   0.143  -0.138   0.424    1.000    0.317

Test for heterogeneity: Q= 185.128 on 13 degrees of freedom (p= 0.000)
Moment-based estimate of between studies variance =  0.241
```

.

B LDL

```
Meta-analysis

       |  Pooled      95% CI         Asymptotic      No. of
Method |     Est   Lower   Upper  z_value  p_value   studies
-------+----------------------------------------------------
Fixed  |   0.219   0.202   0.236   25.397    0.000     14
Random |   0.110   0.038   0.183    2.982    0.003

Test for heterogeneity: Q= 70.416 on 13 degrees of freedom (p= 0.000)
Moment-based estimate of between studies variance =  0.012

Trimming estimator: Linear
Meta-analysis type: Random-effects model

iteration |  estimate    Tn    # to trim     diff
----------+--------------------------------------
    1     |     0.110     53         0         105
    2     |     0.110     53         0           0

Note: no trimming performed; data unchanged

Filled
Meta-analysis

       |  Pooled      95% CI         Asymptotic      No. of
Method |     Est   Lower   Upper  z_value  p_value   studies
-------+----------------------------------------------------
Fixed  |   0.219   0.202   0.236   25.397    0.000     14
Random |   0.110   0.038   0.183    2.982    0.003

Test for heterogeneity: Q= 70.416 on 13 degrees of freedom (p= 0.000)
Moment-based estimate of between studies variance =  0.012

.
```

C HDL

```
Meta-analysis

       |  Pooled      95% CI         Asymptotic      No. of
Method |     Est   Lower   Upper  z_value  p_value   studies
-------+----------------------------------------------------
Fixed  |   0.606   0.278   0.933    3.625    0.000      5
Random |   2.285   0.450   4.119    2.441    0.015

Test for heterogeneity: Q= 111.141 on 4 degrees of freedom (p= 0.000)
Moment-based estimate of between studies variance =  3.990

Trimming estimator: Linear
Meta-analysis type: Random-effects model

iteration |  estimate    Tn    # to trim     diff
----------+--------------------------------------
    1     |     2.285      6         0          15
    2     |     2.285      6         0           0

Note: no trimming performed; data unchanged

Filled
Meta-analysis

       |  Pooled      95% CI         Asymptotic      No. of
Method |     Est   Lower   Upper  z_value  p_value   studies
-------+----------------------------------------------------
Fixed  |   0.606   0.278   0.933    3.625    0.000      5
Random |   2.285   0.450   4.119    2.441    0.015

Test for heterogeneity: Q= 111.141 on 4 degrees of freedom (p= 0.000)
Moment-based estimate of between studies variance =  3.990
```

D UA

Table S7 Trim and fill analysis for the effect of ketogenic diet on TC, LDL, HDL and UA.

(There was no significant difference in heterogeneity and merging results before and after the trim and fill analysis, suggesting that the results were stable.)

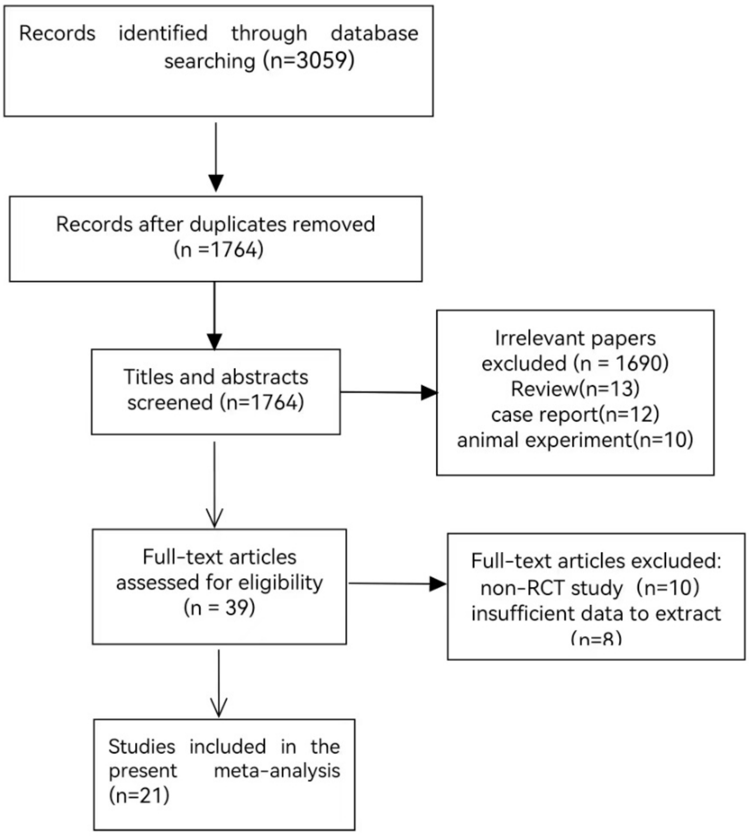

Figure S1 Study selection flow diagram.

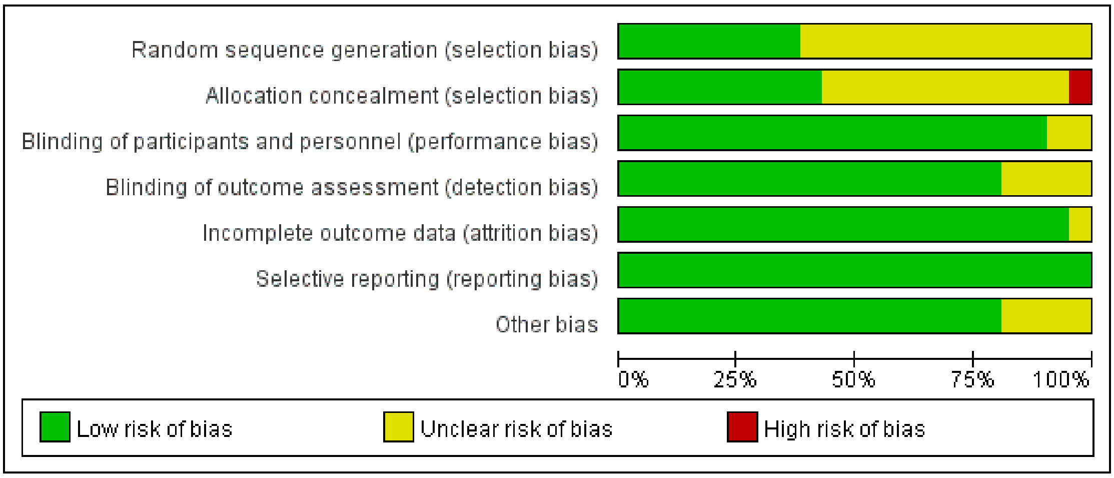

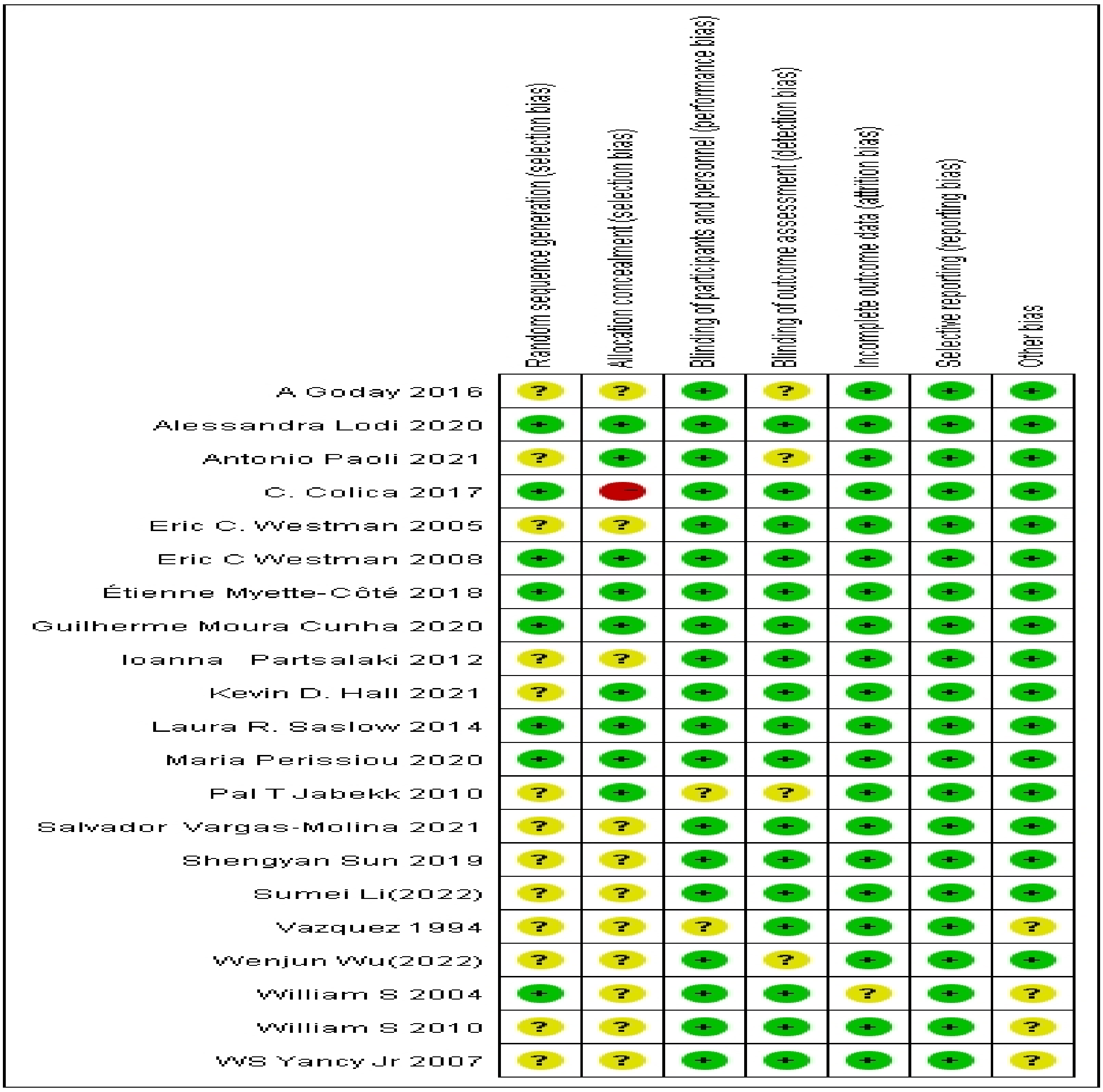

Figure S2 Risk of bias graph and Risk of bias summary

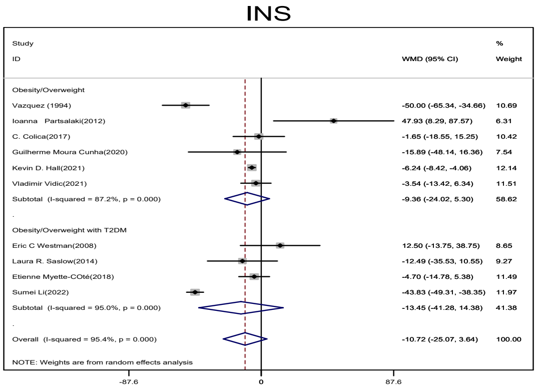

Figure S3 Forest plot detailing weighted mean difference and 95% confidence intervals (CIs) for the effect of ketogenic diet on INS. (Research on sources of heterogeneity not excluded)

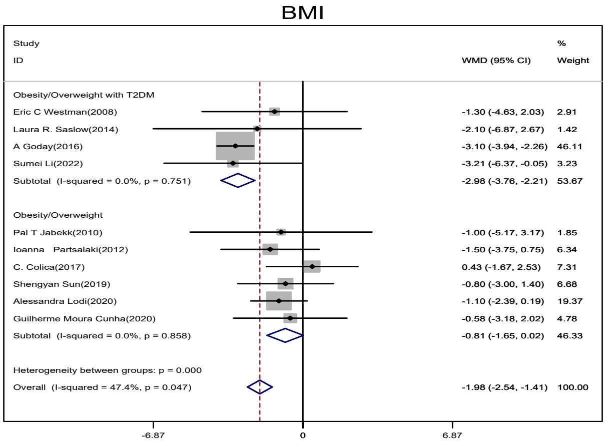

Figure S4 Forest plot detailing weighted mean difference and 95% confidence intervals (CIs) for the effect of ketogenic diet on BMI. (Research on sources of heterogeneity not excluded)

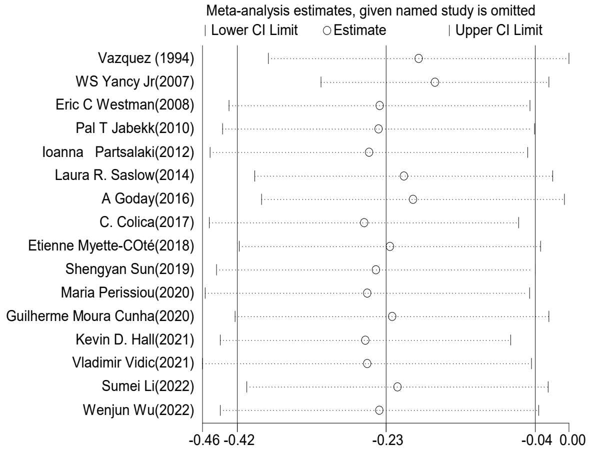

Study omitted	Estimate	[95% Conf.	Interval]
Vazquez (1994)	-.18791956	-.37833887	.00249974
WS Yancy Jr(2007)	-.16733325	-.31169561	-.02297088
Eric C Westman(2008)	-.23753923	-.42798895	-.04708953
Pal T Jabekk(2010)	-.2388722	-.43646023	-.04128417
Ioanna Partsalaki(2012)	-.25092849	-.45203048	-.0498265
Laura R. Saslow(2014)	-.20685628	-.39561439	-.01809819
A Goday(2016)	-.19519112	-.38683283	-.0035494
C. Colica(2017)	-.25707734	-.45298216	-.06117251
Etienne Myette-Cot¨¦(2018)	-.22430569	-.4152095	-.03340188
Shengyan Sun(2019)	-.24201776	-.44399959	-.04003594
Maria Perissiou(2020)	-.2528868	-.4583194	-.04745419
Guilherme Moura Cunha(2020)	-.22152653	-.42022595	-.02282713
Kevin D. Hall(2021)	-.25550157	-.439358	-.07164513
Vladimir Vidic(2021)	-.25326845	-.46157184	-.04496506
Sumei Li(2022)	-.21467845	-.40557408	-.02378283
Wenjun Wu(2022)	-.23760293	-.43936613	-.03583974
Combined	-.22898834	-.417736	-.04024068

A FPG

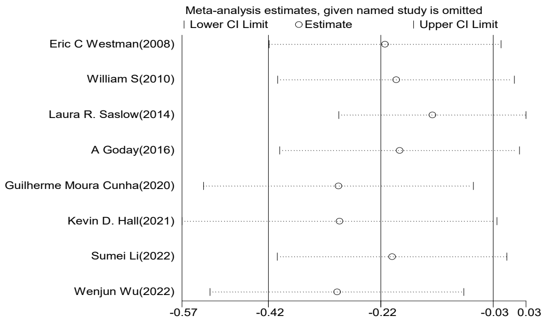

Study omitted	Estimate	[95% Conf.	Interval]
Eric C Westman(2008)	-.21665028	-.41863734	-.01466324
William S(2010)	-.19743893	-.40370911	.00883127
Laura R. Saslow(2014)	-.13420612	-.29748425	.029072
A Goday(2016)	-.191131	-.40012437	.01786239
Guilherme Moura Cunha(2020)	-.29794568	-.53313082	-.06276052
Kevin D. Hall(2021)	-.29619762	-.57076824	-.02162705
Sumei Li(2022)	-.20429899	-.40426728	-.00433069
Wenjun Wu(2022)	-.30046865	-.52183747	-.07909986
Combined	-.22389283	-.41967226	-.0281134

B HbA1c

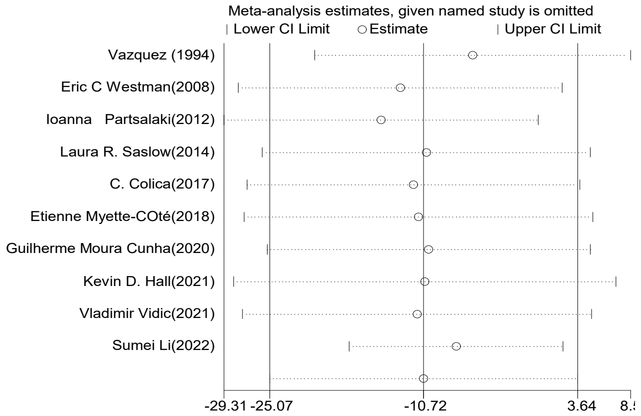

Study omitted	Estimate	[95% Conf.	Interval]
Vazquez (1994)	-6.1550236	-20.87668	8.5666332
Eric C Westman(2008)	-12.89905	-27.981386	2.1832862
Ioanna Partsalaki(2012)	-14.68512	-29.313072	-.05716792
Laura R. Saslow(2014)	-10.469075	-25.751556	4.8134046
C. Colica(2017)	-11.668831	-27.162647	3.824985
Etienne Myette-COt¨¦(2018)	-11.189782	-27.426991	5.0474253
Guilherme Moura Cunha(2020)	-10.255046	-25.305607	4.7955151
Kevin D. Hall(2021)	-10.611458	-28.420912	7.1979952
Vladimir Vidic(2021)	-11.334675	-27.591845	4.9224944
Sumei Li(2022)	-7.6868067	-17.647394	2.2737799
	-10.715712	-25.070478	3.639055
Combined	-10.715711	-25.070478	3.6390549

C INS (Sumei Li's research may be the source of heterogeneity. If this research is excluded from the follow-up research, the heterogeneity will decrease significantly.)

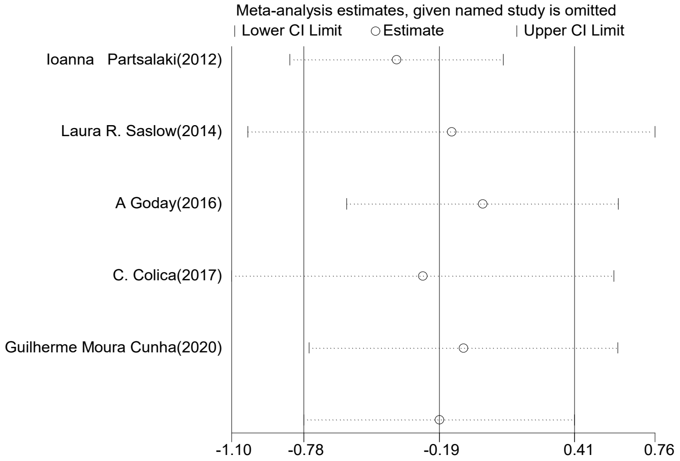

Study omitted	Estimate	[95% Conf.	Interval]
Ioanna Partsalaki(2012)	-.37148738	-.83956099	.09658625
Laura R. Saslow(2014)	-.13109754	-1.0239486	.7617535
A Goday(2016)	.00523677	-.5908435	.60131705
C. Colica(2017)	-.25683534	-1.0951999	.58152926
Guilherme Moura Cunha(2020)	-.0790386	-.75694656	.59886938
	-.18521698	-.77920461	.40877065
Combined	-.18521698	-.77920462	.40877066

D HOMA-IR

Figure S5 Sensitivity analysis for the effect of ketogenic diet on glucose metabolism.

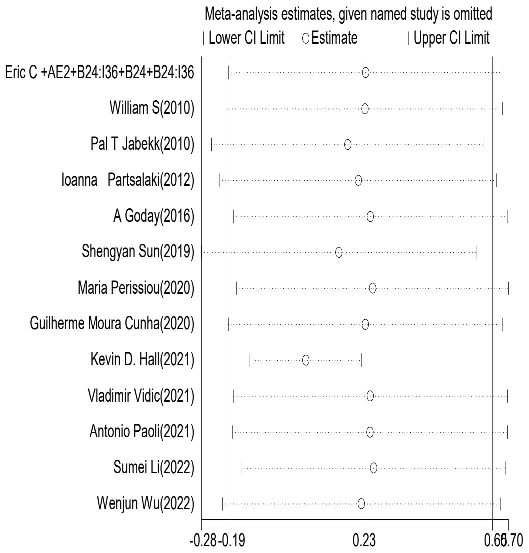

Study omitted	Estimate	[95% Conf.	Interval]
Eric C +AE2+B24:I36+B24+B24:I36	.24275993	-.19460396	.68012381
William S(2010)	.23992762	-.19901644	.67887169
Pal T Jabekk(2010)	.1857197	-.24861608	.6200555
Ioanna Partsalaki(2012)	.21885492	-.22220965	.6599195
A Goday(2016)	.25744221	-.17837675	.69326121
Shengyan Sun(2019)	.1572693	-.28013775	.59467638
Maria Perissiou(2020)	.26485306	-.16798554	.69769168
Guilherme Moura Cunha(2020)	.24141991	-.19496527	.67780513
Kevin D. Hall(2021)	.05178279	-.12603378	.22959936
Vladimir Vidic(2021)	.25734773	-.17943728	.69413275
Antonio Paoli(2021)	.25617489	-.18150991	.6938597
Sumei Li(2022)	.2675488	-.1516387	.68673629
Wenjun Wu(2022)	.22859599	-.21357974	.67077172
Combined	.22816743	-.18951337	.64584823

A TC

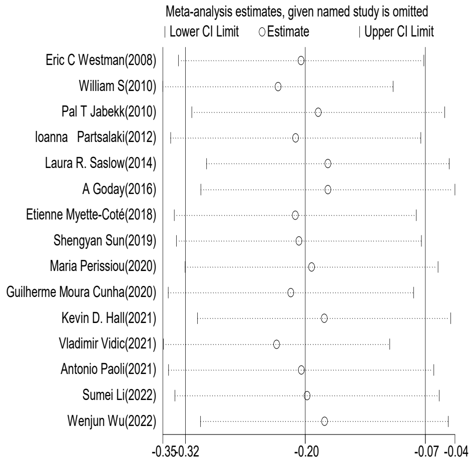

Study omitted	Estimate	[95% Conf.	Interval]
Eric C Westman(2008)	-.19973318	-.32870451	-.07076186
William S(2010)	-.22411217	-.34523979	-.10298456
Pal T Jabekk(2010)	-.18176685	-.31481808	-.04871561
Ioanna Partsalaki(2012)	-.20545745	-.33718789	-.07372701
Laura R. Saslow(2014)	-.1716442	-.29933438	-.04395401
A Goday(2016)	-.17154598	-.30521438	-.0378776
Etienne Myette-Cot¨¦(2018)	-.20595609	-.33321133	-.07870084
Shengyan Sun(2019)	-.20204021	-.33096513	-.07311528
Maria Perissiou(2020)	-.18877438	-.32178259	-.05576617
Guilherme Moura Cunha(2020)	-.21053047	-.33960596	-.081455
Kevin D. Hall(2021)	-.17555328	-.30867681	-.04242975
Vladimir Vidic(2021)	-.22568068	-.34481514	-.10654622
Antonio Paoli(2021)	-.19970065	-.33918428	-.06021702
Sumei Li(2022)	-.19353417	-.3326245	-.05444384
Wenjun Wu(2022)	-.17515099	-.30552348	-.0447785
Combined	-.19527464	-.32146343	-.06908584

B TG

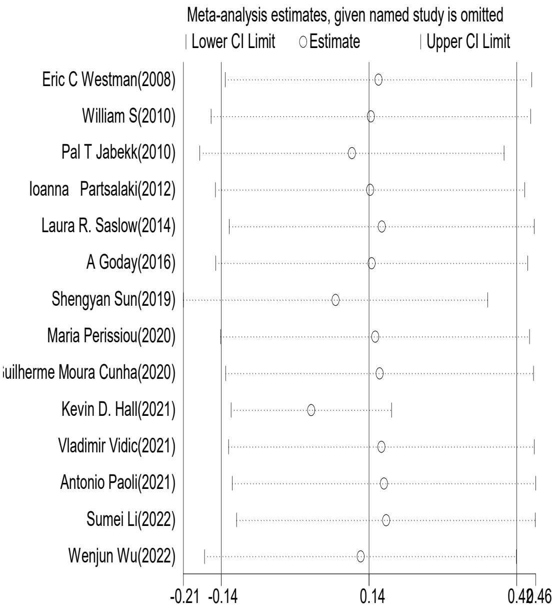

Study omitted	Estimate	[95% Conf.	Interval]
Eric C Westman(2008)	.16107875	-.13061684	.45277435
William S(2010)	.14705904	-.15721364	.4513317
Pal T Jabekk(2010)	.11108111	-.17853771	.40069991
Ioanna Partsalaki(2012)	.14552738	-.1487639	.43981865
Laura R. Saslow(2014)	.16766161	-.12241034	.45773357
A Goday(2016)	.14835633	-.14856727	.44527996
Shengyan Sun(2019)	.07958453	-.20978226	.36895132
Maria Perissiou(2020)	.15479061	-.13937701	.44895825
Guilherme Moura Cunha(2020)	.16327676	-.12983406	.45638758
Kevin D. Hall(2021)	.03340236	-.11937796	.18618268
Vladimir Vidic(2021)	.16695447	-.12425593	.4581649
Antonio Paoli(2021)	.17175712	-.11723118	.46074539
Sumei Li(2022)	.17574845	-.10853433	.46003124
Wenjun Wu(2022)	.1271365	-.16988237	.42415535
Combined	.14333432	-.13766098	.42432961

C LDL

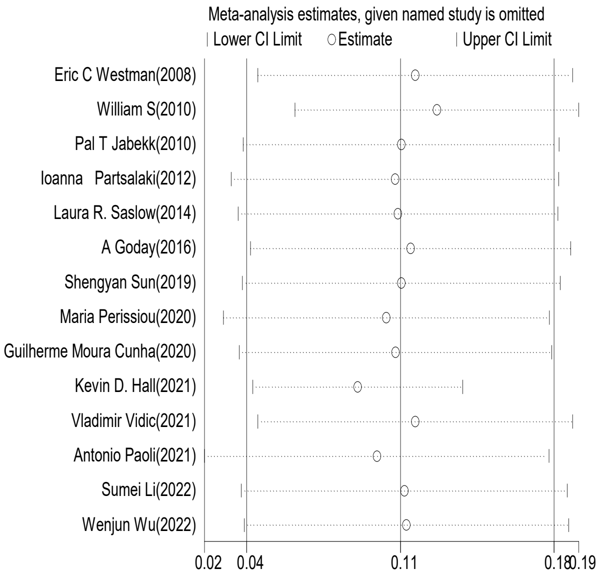

Study omitted	Estimate	[95% Conf.	Interval]
Eric C Westman(2008)	.11737975	.04309729	.19166219
William S(2010)	.12751691	.06051059	.19452325
Pal T Jabekk(2010)	.11070152	.03619768	.18520536
Ioanna Partsalaki(2012)	.1077898	.03053981	.18503979
Laura R. Saslow(2014)	.10921612	.03382958	.18460266
A Goday(2016)	.11512182	.03955286	.19069077
Shengyan Sun(2019)	.11081489	.03583858	.18579119
Maria Perissiou(2020)	.10372157	.02684486	.18059827
Guilherme Moura Cunha(2020)	.1079776	.03424931	.18170589
Kevin D. Hall(2021)	.09023269	.04070818	.13975719
Vladimir Vidic(2021)	.11738374	.04317071	.19159676
Antonio Paoli(2021)	.09919238	.01795674	.18042801
Sumei Li(2022)	.11216676	.03528849	.18904503
Wenjun Wu(2022)	.11322386	.0366159	.18983182
Combined	.11034603	.03780974	.18288231

D HDL

Figure S6 Sensitivity analysis for the effect of ketogenic diet on lipid metabolism.

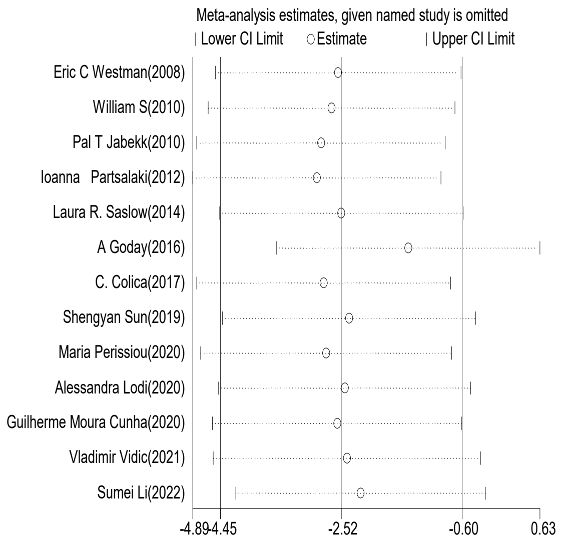

Study omitted	Estimate	[95% Conf.	Interval]
Eric C Westman(2008)	-2.5768397	-4.5307045	-.62297529
William S(2010)	-2.6791024	-4.6403832	-.71782166
Pal T Jabekk(2010)	-2.8458271	-4.8220844	-.86956996
Ioanna Partsalaki(2012)	-2.9147925	-4.8858185	-.94376659
Laura R. Saslow(2014)	-2.5248857	-4.4601278	-.58964348
A Goday(2016)	-1.4611793	-3.5560789	.63372028
C. Colica(2017)	-2.8072603	-4.8260136	-.78850698
Shengyan Sun(2019)	-2.399575	-4.4126596	-.38649052
Maria Perissiou(2020)	-2.7662694	-4.7603855	-.7721535
Alessandra Lodi(2020)	-2.4720564	-4.4789081	-.46520469
Guilherme Moura Cunha(2020)	-2.5918121	-4.5744853	-.60913879
Vladimir Vidic(2021)	-2.4348931	-4.5626769	-.30710927
Sumei Li(2022)	-2.2170432	-4.2020016	-.23208474
Combined	-2.5245253	-4.445705	-.60334567

A Weight

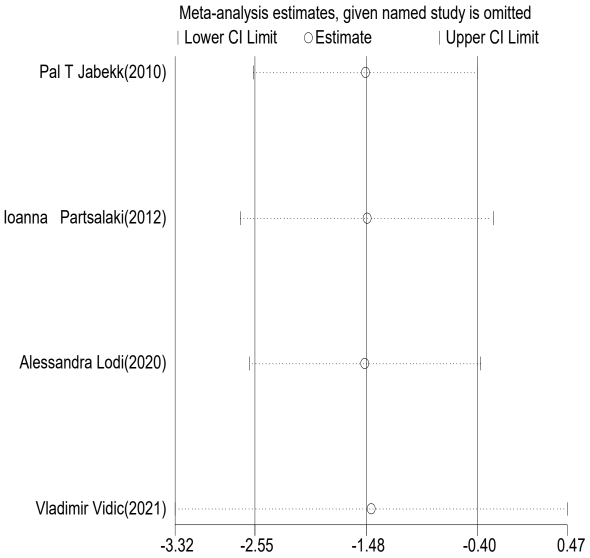

Study omitted	Estimate	[95% Conf.	Interval]
Pal T Jabekk(2010)	-1.4846281	-2.5708268	-.39842936
Ioanna Partsalaki(2012)	-1.4705983	-2.6947258	-.24647075
Alessandra Lodi(2020)	-1.4917489	-2.6105406	-.37295714
Vladimir Vidic(2021)	-1.4295092	-3.3249371	.46591878
Combined	-1.4772922	-2.5530828	-.40150164

B BFM

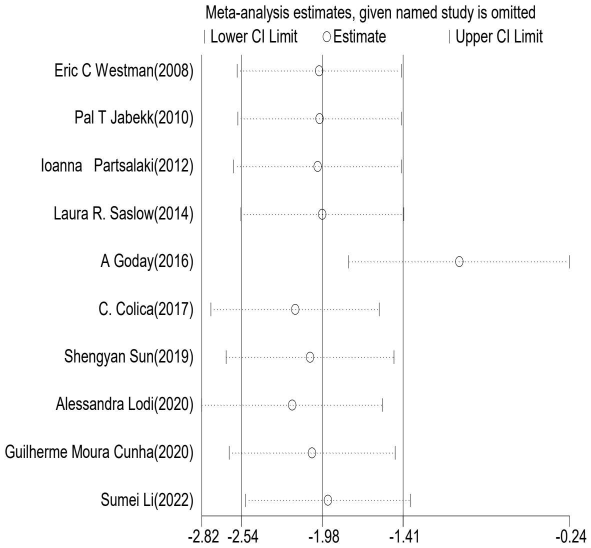

Study omitted	Estimate	[95% Conf.	Interval]
Eric C Westman(2008)	-1.997349	-2.5736039	-1.4210941
Pal T Jabekk(2010)	-1.995501	-2.568645	-1.4223572
Ioanna Partsalaki(2012)	-2.0093689	-2.5960913	-1.4226465
Laura R. Saslow(2014)	-1.975296	-2.5471711	-1.4034209
A Goday(2016)	-1.0160556	-1.789571	-.24254
C. Colica(2017)	-2.1670296	-2.7568214	-1.577238
Shengyan Sun(2019)	-2.0613005	-2.6490788	-1.4735224
Alessandra Lodi(2020)	-2.1877372	-2.8200774	-1.5553972
Guilherme Moura Cunha(2020)	-2.0471575	-2.6290388	-1.4652762
Sumei Li(2022)	-1.9359645	-2.5131614	-1.3587675
Combined	-1.9770621	-2.5448732	-1.409251

C BMI (A Goday's research may be the source of heterogeneity. If this research is excluded from the follow-up research, the heterogeneity will decrease significantly.)

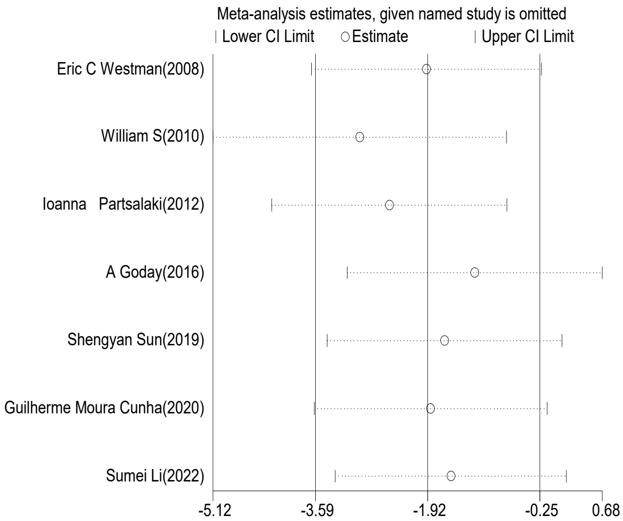

Study omitted	Estimate	[95% Conf.	Interval]
Eric C Westman(2008)	-1.9358268	-3.6483219	-.22333162
William S(2010)	-2.9313219	-5.117918	-.74472606
Ioanna Partsalaki(2012)	-2.4895539	-4.2428412	-.73626661
A Goday(2016)	-1.2176161	-3.1185296	.6832974
Shengyan Sun(2019)	-1.6653783	-3.4163089	.08555229
Guilherme Moura Cunha(2020)	-1.8745085	-3.6085057	-.14051139
Sumei Li(2022)	-1.5727553	-3.2966537	.1511429
Combined	-1.9166422	-3.5881702	-.24511415

D Waistline

Figure S7 Sensitivity analysis for the effect of ketogenic diet on weight control.

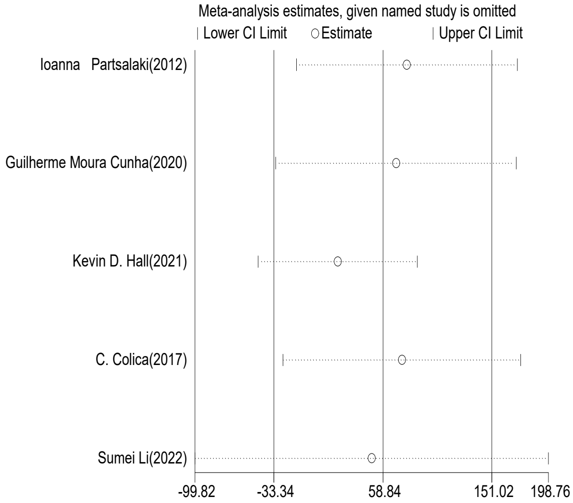

Study omitted	Estimate	[95% Conf.	Interval]
Ioanna Partsalaki(2012)	79.061279	-14.2781	172.40065
Guilherme Moura Cunha(2020)	70.004761	-31.586477	171.59599
Kevin D. Hall(2021)	20.850222	-46.41227	88.112709
C. Colica(2017)	74.989845	-25.415581	175.39526
Sumei Li(2022)	49.471134	-99.816628	198.7589
Combined	58.839608	-33.344531	151.02375

A UA

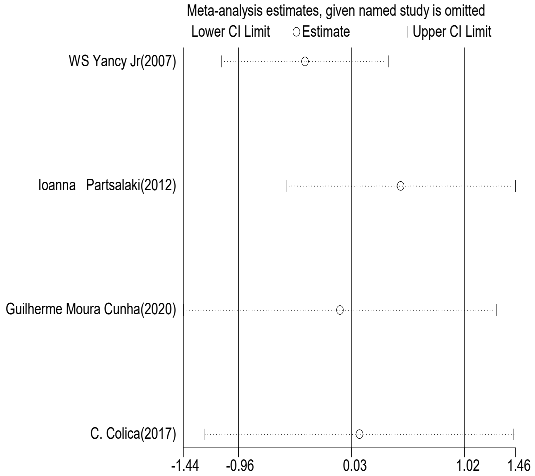

Study omitted	Estimate	[95% Conf.	Interval]
WS Yancy Jr(2007)	-.37747499	-1.1049571	.35000712
Ioanna Partsalaki(2012)	.45814243	-.54347008	1.4597549
Guilherme Moura Cunha(2020)	-.07308499	-1.4367477	1.2905777
C. Colica(2017)	.09627263	-1.2526201	1.4451654
Combined	.02751929	-.95999239	1.015031

B BUN

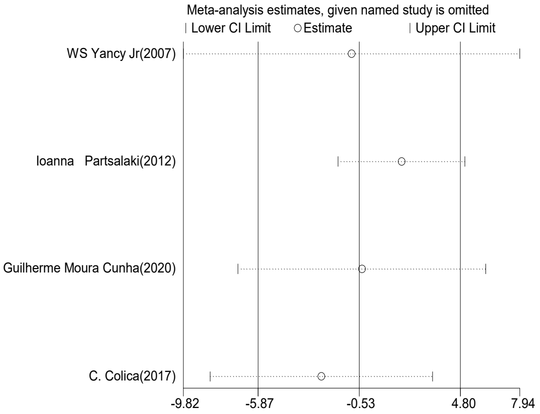

Study omitted	Estimate	[95% Conf.	Interval]
WS Yancy Jr(2007)	-.94107425	-9.820529	7.9383802
Ioanna Partsalaki(2012)	1.692765	-1.6595389	5.0450687
Guilherme Moura Cunha(2020)	-.40252954	-6.9365926	6.1315336
C. Colica(2017)	-2.5411127	-8.4052753	3.3230498
Combined	-.53489622	-5.8734393	4.8036468

C Creatinine

Figure S8 Sensitivity analysis for the effect of ketogenic diet on kidney related functions.

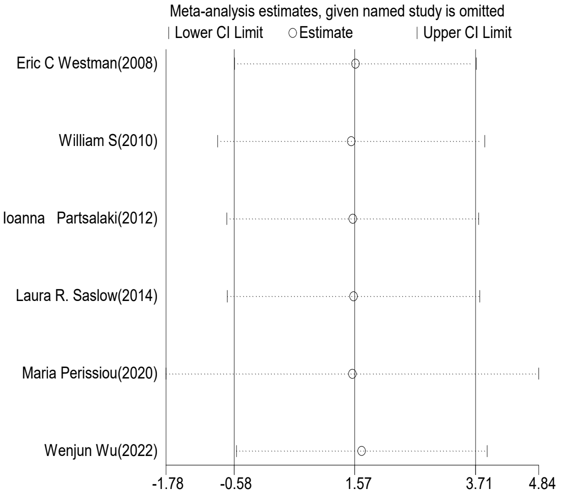

Study omitted	Estimate	[95% Conf.	Interval]
Eric C Westman(2008)	1.5786784	-.56749445	3.7248511
William S(2010)	1.5049239	-.86641347	3.8762615
Ioanna Partsalaki(2012)	1.5326649	-.70202965	3.7673595
Laura R. Saslow(2014)	1.5482413	-.69344378	3.7899263
Maria Perissiou(2020)	1.5275749	-1.7805012	4.8356509
Wenjun Wu(2022)	1.6935583	-.53420824	3.921325
Combined	1.5695627	-.5750997	3.7142251

A SBP

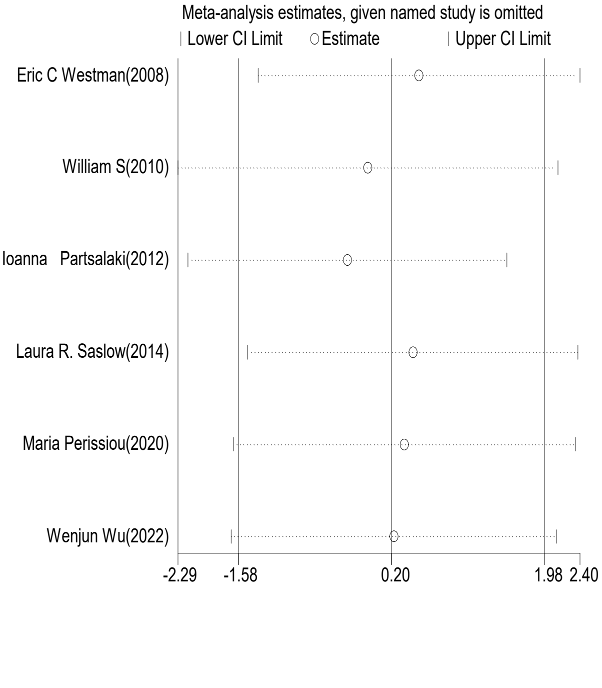

Study omitted	Estimate	[95% Conf.	Interval]
Eric C Westman(2008)	.52076572	-1.3585917	2.4001231
William S(2010)	-.07542846	-2.2921796	2.1413226
Ioanna Partsalaki(2012)	-.31564096	-2.1757886	1.5445068
Laura R. Saslow(2014)	.4489136	-1.4771785	2.3750057
Maria Perissiou(2020)	.34984151	-1.6458269	2.34551
Wenjun Wu(2022)	.22554532	-1.6751904	2.1262813
Combined	.19843263	-1.5844034	1.9812686

B DBP

Figure S9 Sensitivity analysis for the effect of ketogenic diet on blood pressure.
